# Nedosiran in pediatric patients with PH1 and relatively preserved kidney function, a phase 2 study (PHYOX8)

**DOI:** 10.1007/s00467-025-06675-8

**Published:** 2025-01-28

**Authors:** David J. Sas, Sevcan A. Bakkaloglu, Vladimir Belostotsky, Wesley Hayes, Gema Ariceta, Jing Zhou, Verity Rawson

**Affiliations:** 1 Division of Pediatric Nephrology and Hypertension, Mayo Clinic, Rochester, MN USA; 2 Department of Laboratory Medicine and Pathology, Mayo Clinic, Rochester, MN USA; 3 Division of Nephrology and Hypertension, Mayo Clinic, Rochester, MN USA; 4 Department of Pediatric Nephrology, Gazi University, Ankara, Turkey; 5 Department of Pediatrics, McMaster Children’s Hospital, Hamilton, Canada; 6 Department of Pediatric Nephrology, Great Ormond Street Hospital for Children NHS Foundation Trust, London, UK; 7 Pediatric Nephrology, University Hospital Vall d’Hebron, Barcelona, Spain; 8 Novo Nordisk A/S, Lexington, MA USA

**Keywords:** Chronic kidney disease, Gene expression, Hyperoxaluria, Pediatric nephrology, RNAi, Urology

## Abstract

**Background:**

Primary hyperoxaluria type 1 (PH1) is an autosomal recessive disorder with dysregulated glyoxylate metabolism in the liver. Oxalate over-production leads to renal stones, progressive kidney damage and renal failure, with potentially life-threatening systemic oxalosis. Nedosiran is a synthetic RNA interference therapy, designed to reduce hepatic lactate dehydrogenase (LDH) to decrease oxalate burden in PH.

**Methods:**

Currently, in the PHYOX8 study (NCT05001269), pediatric participants (2–11 years) with PH1 (*N* = 15) and estimated glomerular filtration rate (eGFR) ≥ 30mL/min/1.73m^2^ received nedosiran once monthly for 6 months.

**Results:**

Urinary oxalate:creatinine (Uox:Ucr) levels reduced by 64% on average. Mean Uox:Ucr reduction was 52% at day 60 and ˃60% at day 180. At one or more study visits, 93.3% (*N* = 14) of participants reached Uox:Ucr < 1.5 × upper limit of normal (ULN), and 53.3% (*N* = 8) reached ≤ 1.0 × ULN. Median percent change in plasma oxalate (12.0 µmol/L at baseline) to day 180 was –39.23% (*n* = 10). Average number of kidney stones per participant remained stable, whilst a 10.1% average decrease in summed surface area was observed. Median percent change from baseline in eGFR was 2.5%, indicating preservation of renal function.

**Conclusions:**

Nedosiran was well tolerated, with only 3 participants experiencing at least one serious adverse event, none considered treatment-related. The incidence of injection site reactions was 6.7% (1/15 participants). In conclusion, nedosiran treatment led to a significant and sustained reduction of Uox levels in children with PH1. These findings support nedosiran treatment in pediatric patients to reduce Uox and shows promise for limiting PH1-related complications.

**Graphical abstract:**

**Figure:**
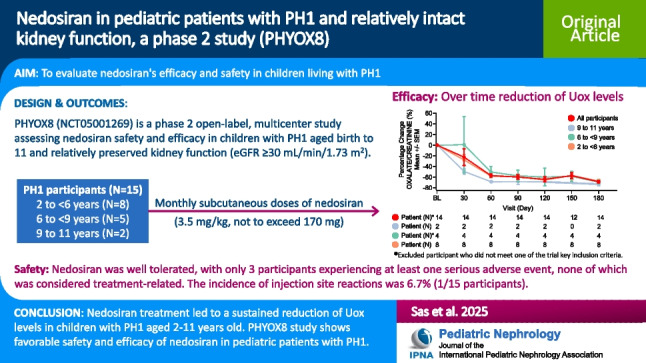
A higher resolution version of the Graphical abstract is available as Supplementary information

**Supplementary Information:**

The online version contains supplementary material available at 10.1007/s00467-025-06675-8.

## Introduction

Primary hyperoxaluria (PH) is a family of 3 rare autosomal-recessive disorders (PH1, PH2, and PH3) each characterized by a specific underlying genetic mutation (*AGXT*, *GRHPR* and, *HOGA1*, respectively) leading to deficiencies in the associated enzymes that are involved in hepatic glyoxylate metabolism [1–3]. Inborn errors of glyoxylate metabolism cause overproduction of endogenous oxalate, a highly insoluble metabolic product, usually eliminated via the kidneys in small amounts [1, 3]. Excess oxalate combines with calcium to form calcium oxalate (CaOx) stones in the kidneys (nephrolithiasis) and reno-urinary tract (urolithiasis). CaOx crystals can deposit in the kidney parenchyma leading to nephrocalcinosis and crystal nephropathy, often resulting in progressive chronic kidney disease (CKD) and kidney failure [1, 3–6]. Kidney function decline is followed by an increase in plasma oxalate (Pox) levels, causing CaOx deposition in various organs, a potentially life-threatening condition known as systemic oxalosis [1, 3, 7–10]. Clinical manifestations of oxalosis include pathological bone fractures, non-healing painful cutaneous ulcers, treatment-refractory anemia, retinopathy, and cardiomyopathy with associated arrhythmias [1, 3, 8]. Historically, liver transplantation has been considered the only potential curative solution to PH-related genetic metabolic deficiency [11–14]. Conventional treatment strategies, including hyperhydration, pyridoxine (vitamin B6), oral inhibitors of CaOx crystallization, and dialysis have typically proven partially effective for long-term control of oxalate levels and are associated with a high treatment burden to the patient [3, 15]. In recent years, the RNA interference (RNAi) therapy lumasiran has been shown to decrease urinary oxalate (Uox) and Pox levels, by reducing glycolate oxidase (GO; encoded by the *HAO1* gene) synthesis in patients living with PH1 [16–18].

Nedosiran, a more recent RNAi therapy, is designed to selectively target the production of hepatic lactate dehydrogenase (LDH; encoded by the *LDHA* gene) [19]. Hepatic LDH plays a central role in the conversion of glyoxylate to oxalate, the final common step in the hepatic glyoxylate metabolism pathway, as the key producer of endogenous oxalate. As such, hepatic LDH is a common therapeutic target across all forms of PH [20–23]. Previous pre-clinical and clinical studies showed that nedosiran-mediated hepatic LDH inhibition was highly selective, with no observed off-target effects [19, 20, 24]. In the phase 1 clinical trial PHYOX1 (NCT03392896), a single dose of nedosiran achieved a significant reduction in Uox excretion in participants with PH1 and PH2 [19]. Currently, nedosiran is approved in the US to lower Uox in children (≥ 9 years) and adults living with PH1 and relatively preserved kidney function (estimated glomerular filtration rate (eGFR) ≥ 30 mL/min/1.73 m^2^) [25].

In PH, excess oxalate burden starts building early in life, with initial symptoms occurring in most individuals before the age of 10 years, and in 85 to 90% by age 20 years [6, 26, 27]. Patients with PH1 in early childhood are at high risk for progressive kidney damage and disease [6]. Primary hyperoxaluria requires early diagnosis and preventive treatment, before clinical symptoms arise and irreversible disease-related complications develop [15]. For these reasons, the phase 2 clinical trial PHYOX8 (NCT05001269) aimed to characterize the efficacy and safety of nedosiran in pediatric participants from 0 to 11 years of age with PH1 and relatively preserved kidney function.

## Methods

### Study design

PHYOX8 (NCT05001269) is a phase 2, multi-dose, open-label, single-arm, uncontrolled, multicenter study assessing nedosiran safety and efficacy in participants from 0 to 11 years of age living with PH and relatively preserved kidney function (see following section for more details). The trial, which is still ongoing, started in February 2022 and by August 2023 15 participants with PH1 (the group for the current interim analysis) had completed the six-month study period. The study was conducted in accordance with the provisions of the Declaration of Helsinki, Good Clinical Practice Guidelines of the International Conference on Harmonization, and all applicable laws and regulations. Written informed consent was obtained from the parents or legal guardians of the participating children. Given the study population being younger than 12 years of age, assent was based on local regulation. Those who completed PHYOX8 were eligible for rolling over into the 6-year open-label extension study PHYOX3 to assess long-term safety and efficacy (NCT04042402).

### Study population

Key inclusion criteria for enrollment were 0 to 11 years of age, genetically confirmed PH1 diagnosis, and relatively preserved kidney function at screening based on eGFR (≥ 30 mL/min normalized to 1.73 m^2^ body surface area (BSA)) for participants over 12 months of age, or serum creatinine (cr) in the normal reference range for participants under 12 months of age where eGFR is not possible. Average spot Uox:Ucr ratio (from 6 screening samples) must have been above 2 times the 95th percentile for age [28].

Key exclusion criteria at screening included prior or planned kidney or hepatic transplantation or dialysis during the study, prior use of an RNAi drug within 6 months, and Pox > 30 μmol/L. Individuals with documented evidence of clinical manifestations of severe systemic oxalosis (including pre-existing retinal, heart, or skin calcifications, or a history of severe bone pain, pathological fractures, or bone deformations) were ineligible. Any participant taking pyridoxine (vitamin B6) must have been on stable dose 3 months prior and throughout study.

One participant was erroneously enrolled in the trial, having inadequately elevated oxalate excretion at screening. This was reported as a protocol deviation, and the deviation was assessed by the Principal Investigator (PI). A sensitivity analysis, which excluded the participant, was also performed and included.

### Interventions

**Fig. 1:**
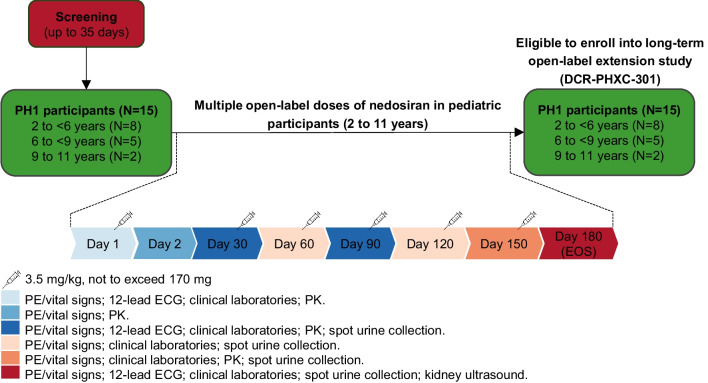
PHYOX8 study overview. Participant overview and study interventions. Physical examination included height/length, weight, inspection of injection site(s), and review of body systems. Vital signs on Day 1 and Day 30 were assessed pre-dose and at 6 h post-dose. On Days 1 and 30, ECG was performed pre-dose and at 6 h post-dose. Clinical laboratory included: hematology (e.g., platelet count), clinical chemistry (e.g., creatinine), coagulation, and urinalysis evaluations. Spot urine samples were collected for the determination of Uox and Ucr. At screening, 6 spot urine samples (3 of which were second morning voids) were collected over a 3-day period. On treatment, 4 spot urine samples (2 of which were second morning voids) were collected over a 2-day period monthly. Two-dimensional surface area of renal stones, measured via kidney ultrasound was performed at baseline and day 180. The kidneys were examined in the longitudinal (sagittal) and transverse scan planes, with images acquired in both the supine and prone positions. A linear array transducer with higher centre frequencies was used. Participants should have had a full bladder during image acquisition. Ultrasound data was transmitted to a standalone central imaging vendor, where qualified personnel performed central overread of all images. **Abbreviations:** *PH1*, primary hyperoxaluria type 1; *PE*, physical examination; *ECG*, electrocardiogram; *PK*, pharmacokinetics; *EOS*, end of study; *Uox*, urinary oxalate; *Ucr*, urinary creatinine

Nedosiran sodium 3.5 mg/kg (not to exceed 170 mg, manufactured by Dicerna Pharmaceuticals, Inc., a Novo Nordisk Company) was administered as a subcutaneous injection into the thigh or abdomen following the schema in Fig. 1. Administration was once a month, for 6 months (end of study, EOS).

Participants avoided vitamin C supplements (including multivitamins) for 24 h prior to urine sample collection, as well as oxalate-rich foods [29]. At discretion of the local nephrologist, all participants were prescribed standard of care for PH, e.g., hyperhydration, oral potassium citrate intake, and treatment with vitamin B6 (if applicable).

### Assessments and endpoints

The primary objective in this study was change from baseline (BL) to month 6 in both absolute and percentage change in spot Uox:Ucr ratio values. The secondary endpoints included efficacy, safety, and pharmacokinetics. Efficacy was measured via *i)* percentage of participants with spot Uox:Ucr ratio ≤ 1.0 × the upper limit of normal (ULN) or ≤ 1.5 × ULN at any time point through month 6, and *ii)* change from BL in eGFR at month 6. Safety was assessed via incidence and nature of any adverse event (AE) and serious adverse event (SAE) reported, along with physical examinations, change from BL in 12-lead electrocardiograms, vital signs, and clinical laboratory tests conducted at screening and throughout the study. Tertiary endpoints included changes in Pox, annualized stone event rate, and kidney stone 2-dimensional summed surface area, which was determined by ultrasound baseline and compared with day 180 scan.

An overview of all procedures and assessments performed in the study, and their timing, is detailed in Fig. 1.

### Statistics

Statistical analysis will be primarily descriptive. Currently, 33 participants have been screened, 12 of those resulted in screen failures, and 21 participants (including all PH types) were enrolled by data cut-off date on Aug 21, 2023. The current sample size of 21 participants (15 PH1) is considered sufficient to provide an assessment of the efficacy and safety of nedosiran in pediatric participants (from 2 to 11 years of age) with PH1 and relatively preserved kidney function. This interim analysis includes the first 15 participants with PH1 who completed the study at data cut-off date.

## Results

### Participants

Out of 33 PH participants screened (ages 2 to 11 years), 15 participants with genetically diagnosed PH1 and relatively preserved kidney function (eGFR ≥ 30 mL/min/1.73 m^2^) had completed the study in this analysis (data cut-off Aug 2023; Fig. 1). None of the 15 participants withdrew from the study, and, at data cut-off, 13 participants continued on to the open-label extension study (PHYOX3; NCT04042402). Participants (66.7% males and 33.3% females) with a median age of 5.0 years, were divided in 3 groups according to their age bracket, children from 2 to < 6 (*N* = 8), 6 to < 9 (*N* = 5), and 9 to 11 (*N* = 2) years old (Table 1). The median age within the 3 groups was 4.0, 7.0, and 9.5. Baseline weight and BSA were within the expected normal parameters for children in each age group. Most of the participants (80%) had either early stage 1 or stage 2 CKD, while 6.7% and 13.3% were affected by stage 3A and stage 3B CKD respectively (Table 1).

**Table 1:** Demographics and Baseline Characteristics

Category or Statistic	Children age group			
	9 to 11 years	6 to < 9 years	2 to < 6 years	Total
	(*N* = 2)	(*N* = 5)	(*N* = 8)	(*N* = 15)
Age (years) [1]				
Mean (SD)	9.5 (0.71)	7.4 (0.55)	3.8 (1.28)	5.7 (2.49)
Median	9.5	7.0	4.0	5.0
Gender, n (%)				
Male	2 (100)	3 (60)	5 (62.5)	10 (66.7)
Female	0	2 (40)	3 (37.5)	5 (33.3)
Race, n (%)				
White	2 (100)	5 (100)	5 ( 62.5)	12 ( 80.0)
Non-White	0	0	3 ( 37.5)	3 ( 20.0)
Weight (kg) [2]				
Mean (SD)	59.50 (16.25)	23.88 (5.88)	16.39 (3.12)	24.63 (15.7)
Median	59.50	21.60	17.00	18.70
Body Surface Area (BSA) (m^2^) [2]				
Mean (SD)	1.465 (0.19)	0.900 (0.15)	0.673 (0.1)	0.854 (0.3)
Median	1,465	0.850	0.680	0.770
eGFR (mL/min/SSA) [2] [3]				
Mean (SD)	86.5 (6.36)	77.0 (17.33)	70.1 (20.75)	74.6 (18.38)
Median	86.5	67.0	72.5	78.0
> = 30 and < 45 mL/min/SSA, n (%)	0	0	1 (12.5)	1 (6.7)
> = 45 mL/min/SSA, n (%)	2 (100)	5 (100)	7 (87.5)	14 (93.3)
Plasma Oxalate (umol/L) [2]				
Mean (SD)	6.500 (6.35)	11.000 (4.85)	14.250 (4.88)	12.133 (5.4)
Median	6,500	12,000	12,500	12,000
Spot Urinary Oxalate (umol/L)				
Mean (SD)	1130.750 (298.4)	751.867 (524.5)	987.333 (557.2)	927.967 (509.05)
Median	1130.750	790,833	882,500	902,667
Spot Urinary Oxalate-to-Creatinine Ratio (umol/mmol)				
Mean (SD)	194.667 (20.02)	285.420 (176.98)	445.742 (195.28)	358.824 (195.28)
Median	194,667	261,600	372,500	303,667
Time since PH Diagnosis (years) [4]				
Mean (SD)	3.867 (0.22)	2.402 (1.72)	1.794 (2.03)	2.273 (1.86)
Median	3,867	3,379	1,420	1,755
Chronic Kidney Disease Stage, n (%)				
Stage 1	2 (100)	2 (40)	2 (25)	6 (40)
Stage 2	0	3 (60)	3 (37.5)	6 (40)
Stage 3 A	0	0	1 (12.5)	1 (6.7)
Stage 3 B	0	0	2 (25)	2 (13.3)
Number of Kidney Stone Events in Last 12 Months [5]				
N	0	4	4	8
Renal and urinary disorders (%)				
Haematuria	0	0	1 (12.5)	1 (6.7)
Vesicoureteric reflux	0	1 (20)	0	1 (6.7)

*SD*, standard deviation; *eGFR*, Estimated Glomerular Filtration Rate; *PH*, Primary Hyperoxaluria; *SSA*, Standard Surface Area

The denominator for the percentage calculation is the number of participants in the analysis population. **[1]** Age in years is collected at the site rather than calculated. **[2]** Baseline is defined as the last non-missing value prior to the first dose of study intervention. BMI is calculated using the following formula: Weight [kg]/(Height^2^ [m^2^]). For spot urinary oxalate and spot urinary oxalate-to-creatinine ratio, baseline is the average of available samples collected at screening. **[3]** eGFR is calculated using the 2009 Schwarz equation for participants < 18 years and Uemura Equation for Japanese participants. **[4]** Time since PH diagnosis (years) is defined as (date of informed consent signature – date of PH diagnosis + 1)/365. PH diagnosis dates with partial dates will be imputed as follows: missing day values will be imputed as 15, and missing month values will be imputed as June. **[5]** Summary only applies to subjects who had a kidney stone event occur in the last 12 months. Kidney stone events in the last 12 months are those events that have a start date within 12 months prior to the date of informed consent signature. Kidney stone event start dates with partial dates will be imputed as follows: missing day values will be imputed as 15, and missing month values will be imputed as June. Concurrent events will be defined as events occurring within the same 4-week (28-day) window

### Efficacy

#### Urinary oxalate:Urinary creatinine ratio

**Fig. 2:**
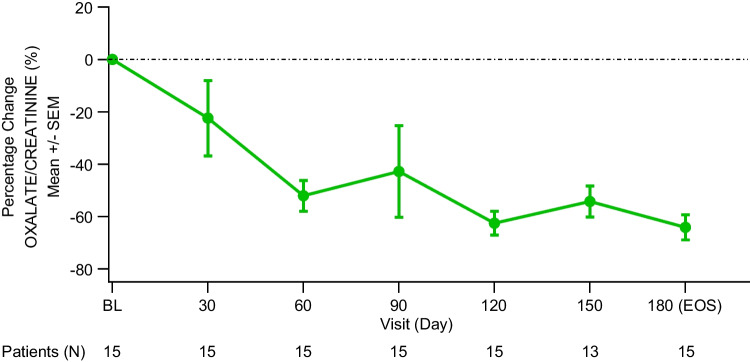
Percent change from baseline in spot Uox:Ucr ratio over time. Patients (N) represent the number of participants assessed at each time point. **Abbreviations**: *SEM*, standard error of the mean; *EOS*, end of study

**Fig. 3:**
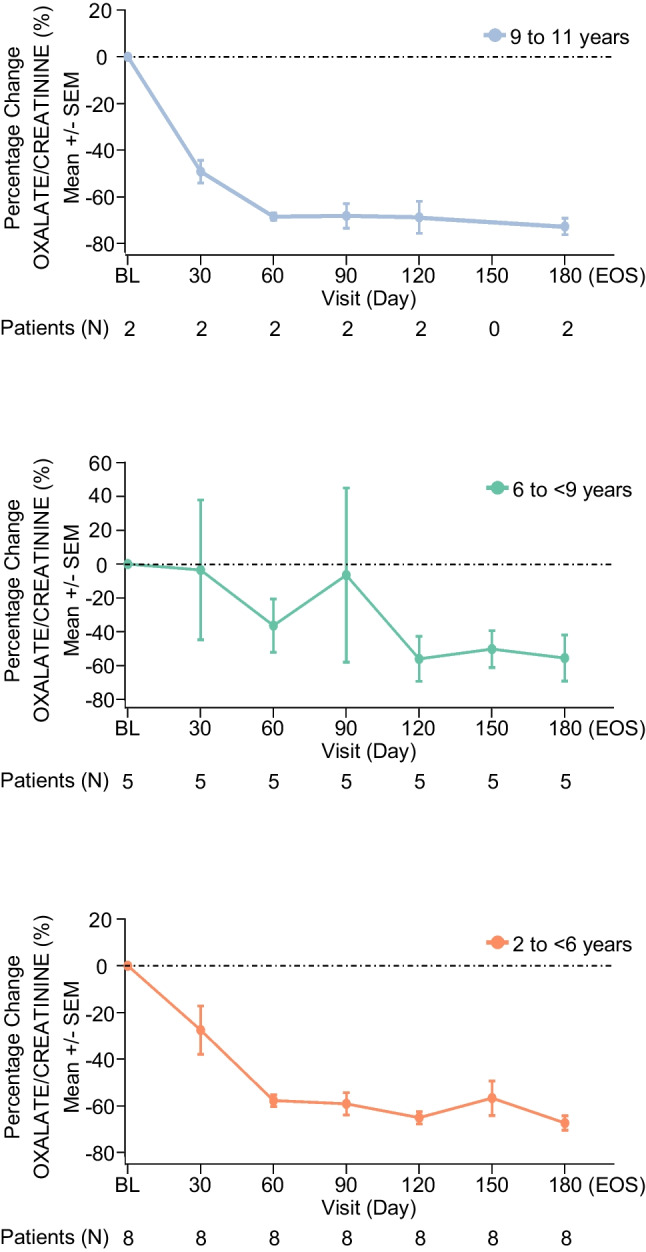
Mean plot for spot Uox:Ucr ratio over time by age group. Patients (N) represent the number of participants assessed at each time point. **Abbreviations:** *SEM*, standard error of the mean; *EOS*, end of study

**Fig. 4:**
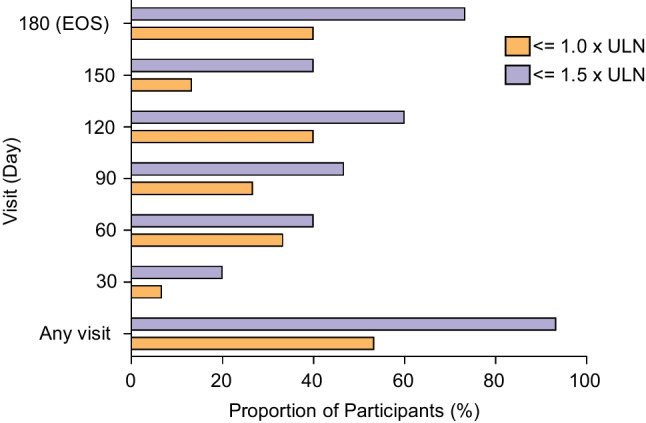
Percentage of participants with ≤ 1.0 × ULN or < 1.5 × ULN of Uox:Ucr excretion throughout the study. Data represent the average of 4 sample/visit. **Abbreviations**: *ULN*, upper limit of normal; *SEM*, standard error of the mean; *EOS*, end of study

PHYOX8 achieved the primary study endpoint by leading to a sustained decrease of Uox:Ucr ratio during the study, showing a rapid decrease at day 30 (by more than 20%), and ˃60% reduction at day 180 (EOS; Fig. 2). The observed Uox:Ucr ratio reduction was comparable between 2 to < 6 and 9 to 11 age groups. Age group 6 to < 9 showed a more gradual Uox decrease, reaching comparable levels to the other two groups at day 120 (Fig. 3). The least squares (LS) mean of percent change from BL to month 6 in Uox:Ucr is –64.1% (95% CI: –83.8, –44.4). When averaged over month 3 to month 6, the LS mean percent change from BL was –56.4% (95% CI: –70.7, –42.1) (Table 2). A sensitivity analysis which excluded 1 participant (from 6 to < 9 group) who did not meet one of the trial key inclusion criteria, who was enrolled in error (having a very low average spot Uox:Ucr ratio at screening; Suppl. Figure 1 and 2), showed a greater reduction in percentage change Uox:Ucr (LS mean –68.5%, 95% Cl: –83.1, –53.9) from BL to month 6 as compared with the primary analysis (Suppl. Table 1). At one or more visits during the study, 93.3% (*N* = 14) of participants with PH1 reached Uox:Ucr less than 1.5 × ULN, and 53.3% (*N* = 8) reached Uox:Ucr ≤ 1.0 × ULN (Fig. 4 and Suppl. Table 2). Specifically, from day 30 to 180, the percentage of participants with ≤ 1.0 × ULN Uox:Ucr increased from 6.7% to 40%, while the percentage with < 1.5 × ULN increased from 20% to 73.3% (Fig. 4 and Suppl. Table 2). The steady improvement in Uox:Ucr ratio, as shown in Fig. 4, appeared to decrease at day 150. However, this was due to a decrease in the number of patients with available data (Suppl. Table 2). Specifically, of the 6 patients with Uox:Ucr ≤ 1.0 × ULN at day 120, 2 still had Uox:Ucr ≤ 1.0 × ULN at day 150, 2 had it less than 1.5 × ULN, and the last 2 (from the 9 to 11 years age group) had missing spot urine data at day 150.

**Table 2:** Change from baseline at month 6 of spot Uox:Ucr ratio results

Category or Statistic	Results
	(*N* = 15)
Baseline [1]	
LS Mean (SE) [2]	360.7 (23)
95% CI for LS Mean [2]	(314.9, 406.3)
Month 6	
LS Mean Percent Change from Baseline (SE) [2]	−64.1 (9.7)
95% CI for Percent Change from Baseline [2]	(−83.8, −44.4)
Averaged over Month 3 to Month 6 [3]	
LS Mean Percent Change from Baseline (SE) [2]	−56.4 (6.6)
95% CI for Percent Change from Baseline [2]	(−70.7, −42.1)

*LS*, Least Squares; *SE*, Standard Error; *CI*, Confidence Interval

**[1]** Baseline spot urine measures calculated as the average of screening results prior to the first dose of study intervention. **[2]** LS Mean and SE are estimated from MMRM model. **[3]** Average estimates based on Day 90, Day 120, Day 150, and Day 180 values

#### Estimated glomerular filtration rate

At BL, eGFR for most of the participants (14/15) was ≥ 45 mL/min/1.73 m^2^, with the exception of 1 participant from the age group of 2 to < 6 who had eGFR between 30 and 45 mL/min/1.73 m^2^ at BL (Table 1). The median percent change in eGFR from BL to month 6 was 2.5%, indicating the preservation of renal function over the course of the study period (Suppl. Table 3).

#### Plasma oxalate

At BL, Pox median was 12.0 µmol/L (Table 1), with only 20% of participants having Pox levels within the normal range (< 10 µmol/L). The median percent change from BL at day 180, measured in 10/15 of the participants, was –39.23% (Suppl. Table 4), with 90% of the participants reaching normal Pox levels and 1 participant remaining near-normal (11 µmol/L).

#### Stone burden

Of the 15 participants, 9 had measurable kidney stones at BL, where a 10.1% average decrease in summed surface area to day 180 was seen (Suppl. Table 5). The average number of stones per participant from BL to day 180 remained stable. Among the participants, 4 (50%) within age group 2 to < 6 years experienced at least one kidney stone event (KSE) in the 12 months prior to screening, and 2 (25%) reported a KSE on study. Four (80%) of those between 6 to < 9 years experienced a KSE 12 months prior to screening, and 2 (40%) experienced a KSE on study. None of the 9 to 11 years group had a recent or on-study KSE (Suppl. Table 5).

### Safety

A total of 53 AEs (6 treatment-related) occurred in 11 out of 15 (73.3%) participants during the study at the time of this interim analysis, none of which led to treatment discontinuation or interruption. Three out of fifteen participants (20%) experienced at least one SAE, for a total of 5 SAEs during the whole study. Specifically, one participant had 2 episodes of nephrolithiasis, one had gastroenteritis and nephrolithiasis, and the third had pyelocaliectasis. None of the SAEs were treatment-related or fatal. One SAE of complex obstructive nephrolithiasis with multiple urinary tract infections (UTI) resulted in acute renal impairment, which slowly improved. The incidence of injection site reactions was 6.7% (1 out of 15 participants). The injection-site reaction was graded as Common Terminology Criteria for Adverse Events (CTCAE) grade 1 and resolved by the end of trial (duration, 2 days). None of the participants experienced either muscle pain or weakness (Table 3). Treatment with nedosiran did not lead to any significant changes in laboratory or other clinical measurements, physical exams, or cardiac assessments. Only 1 out of 15 participants (6.7%) was found positive for anti-drug antibodies (ADA), this was positive from BL (pre-dose) and through day 180, except on day 150 where it was negative (Suppl. Table 6).

**Table 3:** Overall summary of adverse events

	Children age group							
	9 to 11 years		6 to < 9 years		2 to < 6 years		Total	
	(*N* = 2)		(*N* = 5)		(*N* = 8)		(*N* = 15)	
	Number of events	Number (%) of patients with at least one	Number of events	Number (%) of patients with at least one	Number of events	Number (%) of patients with at least one	Number of events	Number (%) of patients with at least one
AEs	0	0	22	5 (100)	31	6 (75)	53	11 (73.3)
Treatment Related AEs	0	0	2	1 (20)	4	2 (25)	6	3 (20)
AEs Leading to Treatment Interruption	0	0	0	0	0	0	0	0
AEs Leading to Treatment Discontinuation	0	0	0	0	0	0	0	0
Serious AEs	0	0	2	1 (20)	3	2 (25)	5	3 (20)
Serious Treatment Related AEs	0	0	0	0	0	0	0	0
Fatal AEs	0	0	0	0	0	0	0	0
AEs of Special Interest [1]	0	0	6	2 (40)	7	3 (37.5)	13	5 (33.3)
Injection Site Reaction [2]								
CTCAE Grade 1	0	0	0	0	1	1 (12.5)	1	1 (6.7)
Muscle Pain or Weakness	0	0	0	0	0	0	0	0
Kidney Stone Events	0	0	6	2 (40)	6	2 (25)	12	4 (26.7)
Mild	0	0	2	2 (40)	2	2 (25)	4	4 (26.7)
Moderate	0	0	0	0	0	0	0	0
Severe	0	0	0	0	0	0	0	0

*AEs*, adverse events; *CTCAE*, common terminology criteria for adverse events

An adverse event (AE) is defined as any adverse event that begins on or after the first dose of study intervention and through the study completion date from the end of study case report form. AEs are considered to be related to study intervention if they are marked as possibly, probably or definitely related to the study intervention on the case report form. AEs are considered as leading to discontinuation if the action taken is marked as “drug withdrawn” on the case report form. AEs are considered to be treated if the action taken is marked as either a new over the counter or prescription drug added, or non-drug therapy on the case report form. AEs are considered to be resolved if the outcome marked is either recovered/resolved or recovered/resolved with sequelae on the case report form. **[1]** AEs of special interest include injection site reactions, muscle pain and weakness, and kidney stone events. **[2]** Injection site reactions (ISR) are defined as signs or symptoms at the injection site with a time to onset of 4 or more hours from the time of study intervention administration

## Discussion

The PHYOX8 study aimed to characterize the efficacy and safety of nedosiran in lowering Uox levels in pediatric participants with PH1 and relatively preserved kidney function. To achieve this, the 15 participants who had completed the study, aged 2 to 11 years at data cut-off date, were treated with nedosiran once a month for 6 months in total. The primary efficacy analysis showed a substantial and consistent reduction in Uox:Ucr throughout the study, with a 64% decrease from BL to day 180 (EOS). Over time, reduction in Uox:Ucr ratio was comparable between the different age groups. Notably, age group 6 to < 9 years exhibited a slower reduction in Uox:Ucr seen at day 90, where one participant had a significantly lower baseline Uox:Ucr. Sensitivity analysis, excluding the above-mentioned participant, demonstrated a greater reduction in percentage change Uox:Ucr compared with the primary analysis encompassing all participants. When evaluating other scenarios where the Uox:Ucr ratio gradually decreases, it is crucial to consider the theory that accelerated bone turnover may play a role. This process could lead to an increase in hydroxyproline, a precursor to oxalate, particularly in children [30, 31].

The proportion of participants achieving ≤ 1.0 × ULN or < 1.5 × ULN Uox:Ucr increased over the course of the study, suggesting an ongoing treatment effect over time. Pox levels normalization in 90% of participants demonstrates the ability of nedosiran to ameliorate the systemic exposure of oxalate in children.

The study also demonstrated that nedosiran treatment resulted in a median percent change in eGFR from baseline to month 6 of 2.5%, highlighting its potential to preserve renal function in children. Additionally, our study observed a 10.1% average decrease in the summed surface area of kidney stones over the six-month period, indicating a promising outcome in reducing the disease burden for young patients.

Nedosiran was well tolerated, with only 3 participants experiencing at least one SAE (nephrolithiasis (3 events), pyelocaliectasis, and gastroenteritis), none of which was considered treatment-related. As for AEs of special interest, no participant suffered muscle pain or weakness, while incidence of injection site reactions and kidney stone related events were 6.7% and 26.7% respectively. The absence of muscle pain supports the lack of nedosiran-related off-target effects. The LDH enzyme is widespread and has critical functions in various tissues [32]. In skeletal muscles, LDH is involved in essential processes for energy production during intense exercise [33]. Inhibiting LDH in skeletal muscles could theoretically lead to muscle pain, weakness, or other adverse effects due to impaired energy metabolism. However, our study, together with previous pre-clinical and clinical studies, showed no evidence of nedosiran-related off-target effects [19, 20, 24, 34, 35].

The AE profile in this study did not reveal any new safety concerns for nedosiran and was in line with previous clinical data in nedosiran [35]. Nine participants exhibited kidney stones at BL, which remained stable. Due to the study’s short duration, changes in number of kidney stones were negligible, with a decrease in burden (summed surface area of kidney stones) of 10.1%. PHYOX8 participant enrolment into the extension study (PHYOX3) will facilitate a more comprehensive long-term analysis of nedosiran impact on kidney stone burden in pediatric patients.

Although nedosiran is thought to not induce or boost ADA [25], one participant was found positive for ADA at BL, before RNAi treatment. It is also known from previous studies, that a small percentage of patients treated with the RNAi therapy lumasiran can develop ADAs [36]. Despite this, in both lumasiran studies and PHYOX8 (data not shown), presence of ADA did not appear to impact efficacy or safety [36].

The results of this 6-month trial demonstrated that nedosiran treatment showed impact at first visit (day 30), leading to a sustained reduction of Uox:Ucr levels from day 60, and was associated with minimal treatment-related AEs in children living with PH1. The impact of nedosiran in reduction of Uox excretion was in line with previous clinical and pre-clinical publications [20]. Kidney function was also preserved in this pediatric cohort overall and stone burden decreased. In conclusion, the results of the PHYOX8 study support the use of nedosiran as a safe and effective treatment for pediatric patients with PH1. Further studies are needed to evaluate the long-term safety and efficacy of nedosiran in this patient population.

## Supplementary Information

Below is the link to the electronic supplementary material.

Graphical abstract (PPTX 272 KB)

Supplementary Figure 1 and 2 (DOCX 35.7 KB)

Supplementary Table 1 (XLSX 18 KB)

Supplementary Table 2 (XLSX 18 KB)

Supplementary Table 3 (XLSX 18.3 KB)

Supplementary Table 4 (XLSX 18 KB)

Supplementary Table 5 (XLSX 20 KB)

Supplementary Table 6 (XLSX 18 KB)

Plain Language Summary (PDF 68 KB)

Ethical Committees (PDF 2664 KB)

## Data Availability

The datasets generated during and/or analyzed during the current study are available from the corresponding author on reasonable request.
