# Supplementary material for: Nedosiran in pediatric patients with PH1 and relatively preserved kidney function, a phase 2 study (PHYOX8)
Source: Pediatr Nephrol. 2025 Jan 28;40(6):1939–48. doi: 10.1007/s00467-025-06675-8 (PMC12031765; doi:10.1007/s00467-025-06675-8)
Supplement: Supplementary file 1 — Graphical abstract (PPTX 272 KB) [file 467_2025_6675_MOESM1_ESM.pptx]

## Slide 1
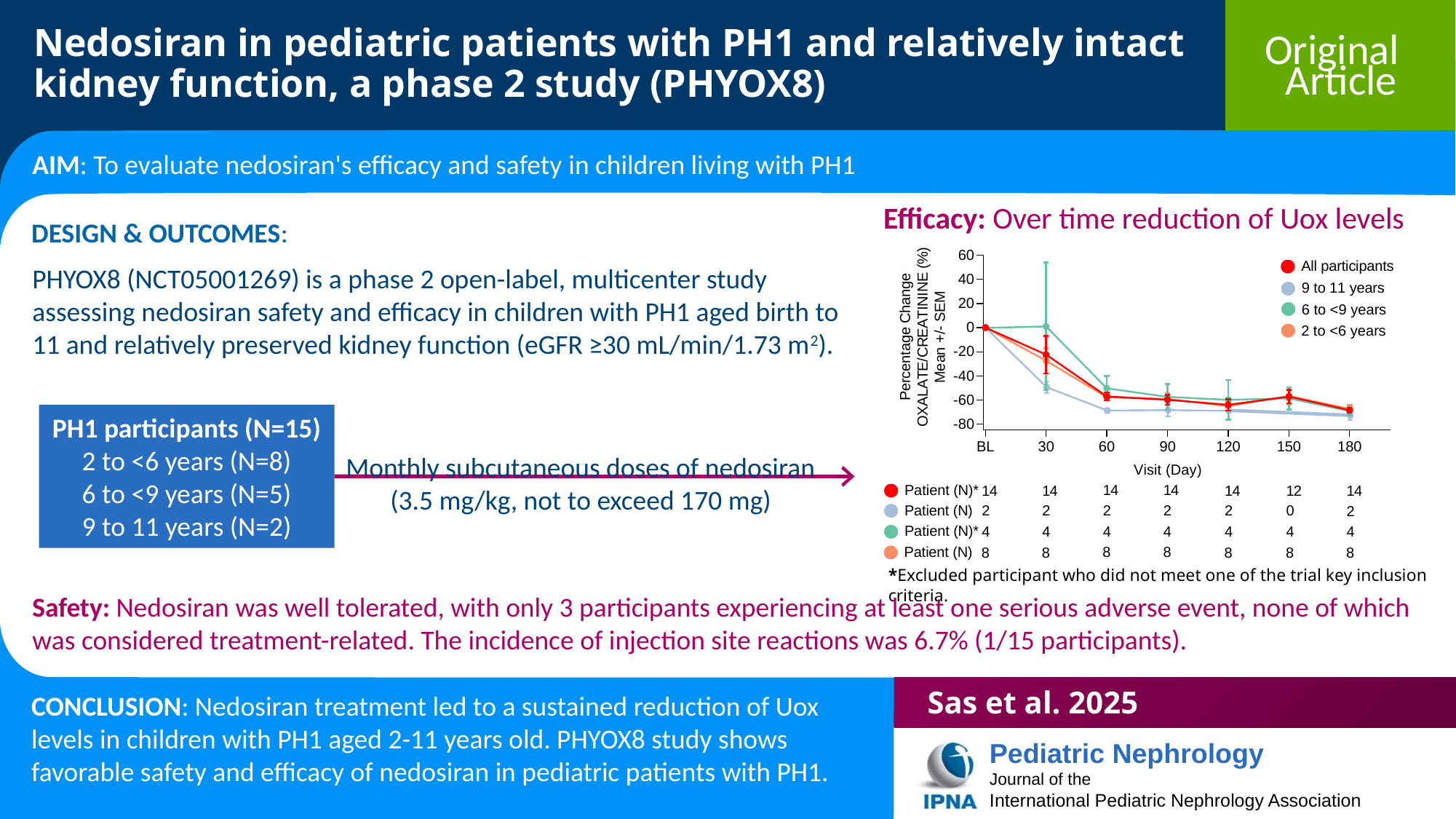

Nedosiran in pediatric patients with PH1 and relatively intact
kidney function, a phase 2 study (PHYOX8)
AIM: To evaluate nedosiran's efficacy and safety in children living with PH1
Efficacy: Over time reduction of Uox levels
DESIGN & OUTCOMES:
*Excluded participant who did not meet one of the trial key inclusion criteria.
PHYOX8 (NCT05001269) is a phase 2 open-label, multicenter study assessing nedosiran safety and efficacy in children with PH1 aged birth to 11 and relatively preserved kidney function (eGFR ≥30 mL/min/1.73 m2).
PH1 participants (N=15)
2 to <6 years (N=8)
6 to <9 years (N=5)
9 to 11 years (N=2)
Monthly subcutaneous doses of nedosiran
(3.5 mg/kg, not to exceed 170 mg)
Safety: Nedosiran was well tolerated, with only 3 participants experiencing at least one serious adverse event, none of which was considered treatment-related. The incidence of injection site reactions was 6.7% (1/15 participants).
Sas et al. 2025
CONCLUSION: Nedosiran treatment led to a sustained reduction of Uox levels in children with PH1 aged 2-11 years old. PHYOX8 study shows favorable safety and efficacy of nedosiran in pediatric patients with PH1.
