# Supplementary material for: Nedosiran in pediatric patients with PH1 and relatively preserved kidney function, a phase 2 study (PHYOX8)
Source: Pediatr Nephrol. 2025 Jan 28;40(6):1939–48. doi: 10.1007/s00467-025-06675-8 (PMC12031765; doi:10.1007/s00467-025-06675-8)
Supplement: Supplementary file 2 — Supplementary Figure 1 and 2 (DOCX 35.7 KB) [file 467_2025_6675_MOESM2_ESM.docx]

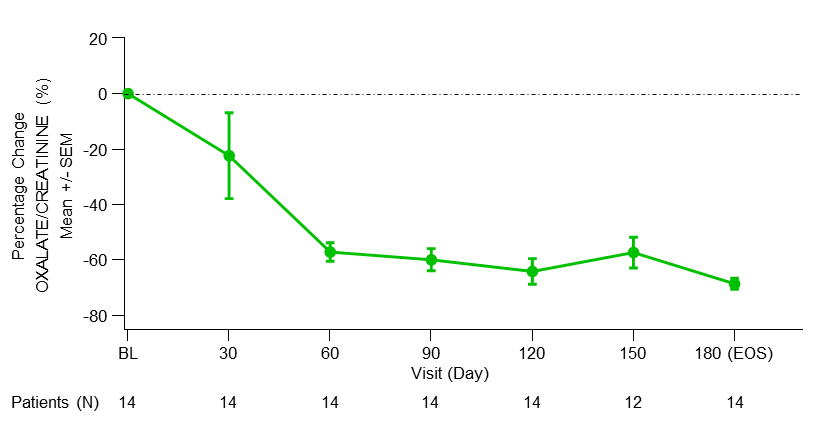


## Suppl. Figure 1. Percent change from baseline in spot Uox:Ucr ratio over time (excluding ineligible participant values)

Patients (N) represent the number of participants assessed at each time point.

**Abbreviations:** SEM, standard error of the mean; EOS, end of study.

##
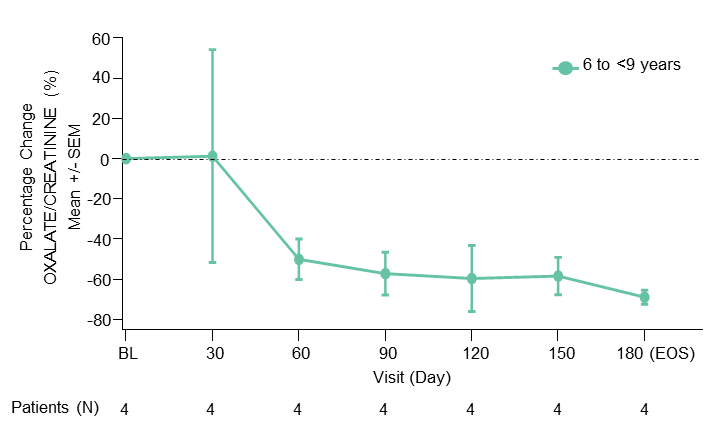
Suppl. Figure 2. Mean plot for spot Uox:Ucr ratio over time 6 to <9 age group (excluding ineligible participant values)

Patients (N) represent the number of participants assessed at each time point.

**Abbreviations:** SEM, standard error of the mean; EOS, end of study.
