# Supplementary material for: Nedosiran in pediatric patients with PH1 and relatively preserved kidney function, a phase 2 study (PHYOX8)
Source: Pediatr Nephrol. 2025 Jan 28;40(6):1939–48. doi: 10.1007/s00467-025-06675-8 (PMC12031765; doi:10.1007/s00467-025-06675-8)
Supplement: Supplementary file 9 — Plain Language Summary (PDF 68 KB) [file 467_2025_6675_MOESM9_ESM.pdf]

PLAIN LANGUAGE SUMMARY

Nedosiran in Pediatric Patients with PH1 and Relatively Preserved Kidney Function, a phase 2 study (PHYOX8)

David J. Sas[1,2,3], Sevcan A. Bakkaloglu[4], Vladimir Belostotsky[5], Wesley Hayes[6], Gema Ariceta[7], Jing Zhou[8], Verity Rawson[8]

Affiliations:
[1]Division of Pediatric Nephrology and Hypertension, Mayo Clinic, Rochester, MN, USA
[2]Department of Laboratory Medicine and Pathology, Mayo Clinic, Rochester, MN, USA.
[3]Division of Nephrology and Hypertension, Mayo Clinic, Rochester, MN, USA.
[4]Department of Pediatric Nephrology, Gazi University, Ankara, Turkey.
[5]Department of Pediatrics, McMaster Children's Hospital, Hamilton, Canada
[6]Department of Pediatric Nephrology, Great Ormond Street Hospital for Children NHS Foundation Trust, London, UK
[7]Pediatric Nephrology, University Hospital Vall d'Hebron, Barcelona, Spain.
[8]Novo Nordisk A/S, Lexington, MA, USA

Once-monthly nedosiran taken for 6 months in children living with primary hyperoxaluria type 1 (PH1)

People living with PH1 are at a higher risk of getting kidney stones and organ damage

- PH1 is a **rare genetic disease** that leads to the build-up of **oxalate**.
- **Oxalate** is a substance made by the body and found in many foods, which is filtered by the kidneys.
- People living with PH cannot eliminate excess oxalate, which can cause **kidney stones** and **kidney failure** over time.
- If PH1 is not treated, oxalate can build up in **other organs**, such as the **bones, eyes**, **heart** and **skin**, leading to serious health issues.

Children with PH1 often experience symptoms before the age of 10, and may already have kidney damage at this young age

It's important to diagnose PH1 early and start treatment early to avoid serious problems

- Nedosiran is a medicine given as a **monthly injection under the skin**.
- Nedosiran works by **reducing the production of oxalate** in the liver.
- PHYOX8 is looking at **how safe and effective nedosiran is for children** (from 0 to 11 years of age) living with PH1.
- Nedosiran treatment in PHYOX8 is tested for **6 months**.

After 6 months, nedosiran reduced the average amount of oxalate in the urine by more than half

- Most of the participants (11 out of 15) experienced **a side-effect**, mild in most cases. **No participant stopped the study because of a side-effect.**
- After 6 months of treatment, **nedosiran reduced** the amount of **oxalate in the urine** and **helped maintain kidney function.**
- Nedosiran is a promising treatment for **reducing oxalate levels** in children with PH1, which could help **prevent kidney damage** and **the formation of kidney stones**.
