# Supplementary material for: Nedosiran in pediatric patients with PH1 and relatively preserved kidney function, a phase 2 study (PHYOX8)
Source: Pediatr Nephrol. 2025 Jan 28;40(6):1939–48. doi: 10.1007/s00467-025-06675-8 (PMC12031765; doi:10.1007/s00467-025-06675-8)
Supplement: Supplementary file 10 — Ethical Committees (PDF 2664 KB) [file 467_2025_6675_MOESM10_ESM.pdf]

9.1.3 List of independent ethics committees and-or institutional review boards

List of contents

Ethics committee list - AE .... Link
Ethics committee list - CA .... Link
Ethics committee list - DE .... Link
Ethics committee list - ES .... Link
Ethics committee list - GB .... Link
Ethics committee list - IT .... Link
Ethics committee list - JP .... Link
Ethics committee list - LB .... Link
Ethics committee list - PL .... Link
Ethics committee list - TR .... Link
Ethics committee list - US .... Link
Master participant information/informed consent child .... Link
Master participant information/informed consent adolescent .... Link
Master participant information/informed consent parent .... Link

List of Independent Ethics Committees/Institutional Review Boards

The study protocol and any subsequent protocol versions and/or applicable protocol amendments were submitted for review, according to local requirements, by an independent ethics committee (IEC)/institutional review board (IRB), as shown below. The IEC/IRB was comprised of a review panel responsible for ensuring the protection of the rights, safety, and well-being of human subjects involved in a clinical investigation. The IEC/IRB was adequately constituted to provide assurance of that protection according to the requirements of ICH GCP.

Site no.	Investigator name<br>Address	IEC/IRB Name<br>IEC/IRB Address	Protocol versions		Protocol amendments	
			Number	IEC/IRB approval date	Number	IEC/IRB approval date
940	Loai Eid<br>Abu Baker Al Siddique Road<br>PO Box 4545<br>Dubai<br>United Arab Emirates	Dubai Scientific Research Ethics Committee (DSREC)<br>Dubai Health Authority<br>Dubai<br>United Arab Emirates	1.0	NA	4.0	30-Nov-2023

List of Independent Ethics Committees/Institutional Review Boards

The study protocol and any subsequent protocol versions and/or applicable protocol amendments were submitted for review, according to local requirements, by an independent ethics committee (IEC)/institutional review board (IRB), as shown below. The IEC/IRB was comprised of a review panel responsible for ensuring the protection of the rights, safety, and well-being of human subjects involved in a clinical investigation. The IEC/IRB was adequately constituted to provide assurance of that protection according to the requirements of ICH GCP.

**Site no.**	**Investigator name**<br>**Address**	**IEC/IRB Name**<br>**IEC/IRB Address**	**Protocol versions**		**Protocol amendments**	
			**Number**	**IEC/IRB approval date**	**Number**	**IEC/IRB approval date**
150	Vladimir Belostotsky<br>1280 Main Street West<br>Hamilton, Ontario L8S 4K1<br>Canada	Hamilton Integrated Research Ethics Board<br>237 Barton Street East<br>Hamilton, Ontario L8L 2X2<br>Canada	1.0	17-Jan-2022	2.0<br>3.0<br>4.0	15-Aug-2022<br>21-Mar-2023<br>18-Jul-2023

List of Independent Ethics Committees/Institutional Review Boards

The study protocol and any subsequent protocol versions and/or applicable protocol amendments were submitted for review, according to local requirements, by an independent ethics committee (IEC)/institutional review board (IRB), as shown below. The IEC/IRB was comprised of a review panel responsible for ensuring the protection of the rights, safety, and well-being of human subjects involved in a clinical investigation. The IEC/IRB was adequately constituted to provide assurance of that protection according to the requirements of ICH GCP.

**Site no.**	**Investigator name**<br>**Address**	**IEC/IRB Name**<br>**IEC/IRB Address**	**Protocol versions**		**Protocol amendments**	
			**Number**	**IEC/IRB approval date**	**Number**	**IEC/IRB approval date**
421	Prof. Burkhard Tönshoff<br>Im Neuenheimer Feld 430<br>Heidelberg 69120<br>Germany	Heidelburg University Ethics Committee of the Med. Faculty<br>Alte Glockengießerei 11/1<br>69115 Heidelberg<br>Germany	1.0	4-Oct-2021	2.0<br>4.0	19-Apr-2022<br>02-Aug-2023
423	Dr. Gesa Schalk<br>Im Mühlenbach 2b<br>Bonn 53127<br>Germany	Heidelburg University Ethics Committee of the Med. Faculty<br>Alte Glockengießerei 11/1<br>69115 Heidelberg<br>Germany	1.0	4-Oct-2021	2.0<br>4.0	19-Apr-2022<br>02-Aug-2023

List of Independent Ethics Committees/Institutional Review Boards

The study protocol and any subsequent protocol versions and/or applicable protocol amendments were submitted for review, according to local requirements, by an independent ethics committee (IEC)/institutional review board (IRB), as shown below. The IEC/IRB was comprised of a review panel responsible for ensuring the protection of the rights, safety, and well-being of human subjects involved in a clinical investigation. The IEC/IRB was adequately constituted to provide assurance of that protection according to the requirements of ICH GCP.

Site no.	Investigator name<br>Address	IEC/IRB Name<br>IEC/IRB Address	Protocol versions		Protocol amendments	
			Number	IEC/IRB approval date	Number	IEC/IRB approval date
312	Gema Ariceta<br>Paseo Vall d'Hebron, 119-129<br>Barcelona 08035<br>Spain	Hospital Germans Trias i Pujol<br>Carretera de Canyet, s/n, 08916 Badalona, Barcelona<br>Spain	1.0	21-Jul-2021	2.0<br>3.0<br>4.0	12-Apr-2022<br>24-Feb-2023<br>18-Jul-2023

List of Independent Ethics Committees/Institutional Review Boards

The study protocol and any subsequent protocol versions and/or applicable protocol amendments were submitted for review, according to local requirements, by an independent ethics committee (IEC)/institutional review board (IRB), as shown below. The IEC/IRB was comprised of a review panel responsible for ensuring the protection of the rights, safety, and well-being of human subjects involved in a clinical investigation. The IEC/IRB was adequately constituted to provide assurance of that protection according to the requirements of ICH GCP.

**Site no.**	**Investigator name**<br>**Address**	**IEC/IRB Name**<br>**IEC/IRB Address**	**Protocol versions**		**Protocol amendments**	
			**Number**	**IEC/IRB approval date**	**Number**	**IEC/IRB approval date**
444	Wesley Hayes<br>Level 1 Frontage Building Great Ormond Street<br>London WC1N 3JH<br>England	South Central - Hampshire A Research Ethics Committee<br>Temple Quay House<br>2 The Square<br>Bristol BS1 6PN<br>England	1.0	23-Aug-2021	2.0<br>4.0	25-Apr-2022<br>7-Aug-2023

VV-CLIN-228645 1.0

List of Independent Ethics Committees/Institutional Review Boards

The study protocol and any subsequent protocol versions and/or applicable protocol amendments were submitted for review, according to local requirements, by an independent ethics committee (IEC)/institutional review board (IRB), as shown below. The IEC/IRB was comprised of a review panel responsible for ensuring the protection of the rights, safety, and well-being of human subjects involved in a clinical investigation. The IEC/IRB was adequately constituted to provide assurance of that protection according to the requirements of ICH GCP.

**Site no.**	**Investigator name**<br>**Address**	**IEC/IRB Name**<br>**IEC/IRB Address**	**Protocol versions**		**Protocol amendments**	
			**Number**	**IEC/IRB approval date**	**Number**	**IEC/IRB approval date**
321	Francesco Emma<br>Piazza di Sant'Onofrio, 4<br>Rome 00165<br>Italy	Ospedale Pediatrico Bambino Gesu<br>Piazza S. Onofrio, 4<br>Rome 00163<br>Italy	1.0	05-Aug-2021	2.0<br>3.0<br>4.0	08-Jul-2022<br>19-Oct-2022<br>15-May-2023

List of Independent Ethics Committees/Institutional Review Boards

The study protocol and any subsequent protocol versions and/or applicable protocol amendments were submitted for review, according to local requirements, by an independent ethics committee (IEC)/institutional review board (IRB), as shown below. The IEC/IRB was comprised of a review panel responsible for ensuring the protection of the rights, safety, and well-being of human subjects involved in a clinical investigation. The IEC/IRB was adequately constituted to provide assurance of that protection according to the requirements of ICH GCP.

Site no.	Investigator name<br>Address	IEC/IRB Name<br>IEC/IRB Address	Protocol versions		Protocol amendments	
			Number	IEC/IRB approval date	Number	IEC/IRB approval date
803	Shuzo Hamamoto<br>1, Kawasumi, Mizuho-cho<br>Mizuho-ku<br>Nagoya 467-8601<br>Japan	**Local:** Nagoya City University Institutional Review Board<br>1, Kawasumi, Mizuho-cho,<br>Mizuho-ku,<br>Nagoya-shi<br>Japan	1.0	NA	2.0<br>4.0	02-Jun-2022<br>07-Aug-2023
804	Seiji Tanaka<br>67 Asahi-machi<br>Kurume-shi<br>Fukuoka<br>Japan	Institutional Review Board<br>Kurume University Hospital<br>Clinical Trial Review Board<br>67 Asahi-machi<br>Kurume-shi<br>Fukuoka<br>Japan	1.0	NA	2.0<br>3.0<br>4.0	20-Apr-2022<br>20-Jul-2023<br>24-Aug-2023

.

List of Independent Ethics Committees/Institutional Review Boards

The study protocol and any subsequent protocol versions and/or applicable protocol amendments were submitted for review, according to local requirements, by an independent ethics committee (IEC)/institutional review board (IRB), as shown below. The IEC/IRB was comprised of a review panel responsible for ensuring the protection of the rights, safety, and well-being of human subjects involved in a clinical investigation. The IEC/IRB was adequately constituted to provide assurance of that protection according to the requirements of ICH GCP.

Site no.	Investigator name<br>Address	IEC/IRB Name<br>IEC/IRB Address	Protocol versions		Protocol amendments	
			Number	IEC/IRB approval date	Number	IEC/IRB approval date
911	Chebl Mourani<br>Blvd Alfred Naccache Achrafieh<br>Beirut<br>Lebanon	Comité d'éthique<br>Hôtel-Dieu de France<br>B.P. 16-6830 Achrafieh<br>Beirut<br>Lebanon	1.0	08-Jul-2021	2.0<br>3.0<br>4.0	04-Apr-2022<br>27-Feb-2023<br>24-Jul-2023

List of Independent Ethics Committees/Institutional Review Boards

The study protocol and any subsequent protocol versions and/or applicable protocol amendments were submitted for review, according to local requirements, by an independent ethics committee (IEC)/institutional review board (IRB), as shown below. The IEC/IRB was comprised of a review panel responsible for ensuring the protection of the rights, safety, and well-being of human subjects involved in a clinical investigation. The IEC/IRB was adequately constituted to provide assurance of that protection according to the requirements of ICH GCP.

Site no.	Investigator name<br>Address	IEC/IRB Name<br>IEC/IRB Address	Protocol versions		Protocol amendments	
			Number	IEC/IRB approval date	Number	IEC/IRB approval date
411	Anna Wasilewska MD<br>ul. Jerzego Waszyngtona 17<br>Bialystok 15-274<br>Poland	Białystok Medical University<br>Jana Kilińskiego 1<br>Białystok 15-089<br>Poland	1.0	24-Jun-2021	2.0<br>3.0<br>4.0	28-Apr-2022<br>30-Mar-2023<br>22-Jun-2023

.

List of Independent Ethics Committees/Institutional Review Boards

The study protocol and any subsequent protocol versions and/or applicable protocol amendments were submitted for review, according to local requirements, by an independent ethics committee (IEC)/institutional review board (IRB), as shown below. The IEC/IRB was comprised of a review panel responsible for ensuring the protection of the rights, safety, and well-being of human subjects involved in a clinical investigation. The IEC/IRB was adequately constituted to provide assurance of that protection according to the requirements of ICH GCP.

Site no.	Investigator name<br>Address	IEC/IRB Name<br>IEC/IRB Address	Protocol versions		Protocol amendments	
			Number	IEC/IRB approval date	Number	IEC/IRB approval date
920	Dr. Sevcan Bakkaloglu<br>Emniyet Neighborhood,<br>Mevlana Boulevard, 29<br>Yenimahalle, Ankara 06560<br>Turkey	Gazi University Medical Faculty Clinical Trials Ethics Committee<br>Gazi University Medical Faculty<br>Dean’s Building 06500<br>Beşevler/Ankara<br>Turkey	1.0	29-Nov-2021	2.0<br>3.0<br>4.0	18-Apr-2022<br>19-Jun-2023<br>19-Jun-2023

.

List of Independent Ethics Committees/Institutional Review Boards

The study protocol and any subsequent protocol versions and/or applicable protocol amendments were submitted for review, according to local requirements, by an independent ethics committee (IEC)/institutional review board (IRB), as shown below. The IEC/IRB was comprised of a review panel responsible for ensuring the protection of the rights, safety, and well-being of human subjects involved in a clinical investigation. The IEC/IRB was adequately constituted to provide assurance of that protection according to the requirements of ICH GCP.

Site no.	Investigator name<br>Address	IEC/IRB Name<br>IEC/IRB Address	Protocol versions		Protocol amendments	
			Number	IEC/IRB approval date	Number	IEC/IRB approval date
101	Dr. David Sas<br>200 First St SW<br>Rochester, Minnesota 55905	WCG IRB<br>1019 39th Ave SE #120<br>Puyallup, WA 98374	1.0	27-Aug-2021	2.0<br>3.0<br>4.0	27-Apr-2022<br>05-Sep-2023<br>24-Feb-2024

Assent Form for Children

Recommended for ages 6 to 11 years

Depending on the reading level of the child, this form can be shown/read to the child by his or her parent/guardian.

Study Title: A Phase 2 Open-Label Multicenter Study to Evaluate the Efficacy, Safety, and Pharmacokinetics of Nedosiran in Pediatric Patients from Birth to 11 Years of Age with Primary Hyperoxaluria and Relatively Intact Renal Function

**Principal Investigator:**	Investigator Name	Telephone: (xxx) xxx-xxxx
**Emergency Contact:**	Name	Telephone: (xxx) xxx-xxxx
**Available 24 hours a day:**	*[Should be a phone number accessible 24/7 and answered by someone knowledgeable about the study]*	

Participant age: _______________ years.

Should the assenting child decide not to take part in this study, the parent/guardian may not force the child to take part.

We are asking if you would like to help us do a study. A study is a test and/or question that can help us learn new things. In this study, we want to test a new drug. By asking you and other people like you if they want to help with our study, we might be able to answer some questions we have about people with primary hyperoxaluria. Primary hyperoxaluria might be written as 'PH' on the other pages of this form. Because you have PH, we are asking you if you want to be in this study. You do not have to do the study if you do not want to.

You can ask your study doctor to tell you about any of the words on these pages or anything about the study that you do not understand. You can ask as many questions as you like any time you think of them. You can also talk about the study with your family and friends if you want to. Please take your time thinking about if you want to do the study or not.

This form tells you important things about the study. It will tell you why we are doing the study and if something good or something bad could happen to you during the study.

You do not have to do the study if you do not want to. If you decide to do the study and then change your mind, you can stop doing the study any time. No one will be upset with you if you decide to stop doing the study or if you do not want to do the study at all.

Why are we asking you to do the study?

You are being asked to take part in this study because you have PH, and your kidneys do not work as well as they could. We would like to give you the study drug, take some blood, do some tests, and ask you things that will tell us more about people with PH. Your study doctor will tell you and your mom, dad, or the person who takes care of you about the study.

The study doctor will explain why the study is being done, and also what you and your family would have to do if you want to be in the study.

We would like to talk about the study with you and your mom, dad, or the person who takes care of you, so you can choose if you want to do the study or not. Whatever you decide is okay.

Do you have to be in the study?

No; you do not have to be in the study if you do not want to. It is up to you. If you decide to do the study and then later change your mind, you can stop any time. No one will be upset with you.

What will happen if you do the study?

If you want to do the study, you will need to have some tests done. These tests may be done at the doctor's office or at home. You will need these tests every month for 7 months. There will be some telephone calls too, where the study doctor will ask some questions about how you are feeling and any medicines you are taking. All tests will be explained to you by the doctor, your mom, dad, or the person who takes care of you before they are done. Some of these tests will be done many times.

You will be given the study drug every month. The study drug is given with a needle under the skin in your upper leg or tummy. You will be given the study drug by the study doctor, a nurse, or your mom, dad, or the person who takes care of you at the doctor's office or at home.

During this study, the study doctor (or a nurse who will visit your home) will:

- Give you a check-up, see how tall you are, and how much you weigh
- Ask your mom, dad, or the person who takes care of you about your family background, including your ethnicity (where your family is from)
- Ask what medicines you are taking
- Ask you about how PH has affected you
- Check to see how your heart is working
- Collect some of your blood
- Check that you have PH – the doctor or nurse might rub a small soft stick (like a cotton swab or Q-tip) on the inside of your cheek

- Collect some of your urine (pee) in a cup
- Ask questions about how you feel and whether anything hurts
- Check your blood pressure, breathing, and heart rate
- Do an ultrasound (jelly scan) of your tummy and/or heart. The doctor or nurse will put gel on your tummy or chest and use a wand to take pictures
- Give you the study drug and/or teach your mom, dad, or the person who takes care of you how to give you the study drug, and watch them do it to make sure they are doing it right

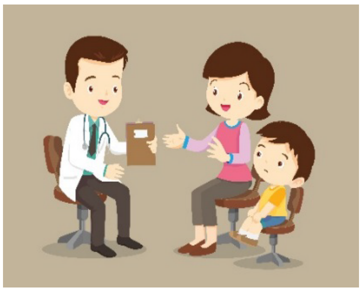

The doctor or nurse will ask you about how you are feeling.

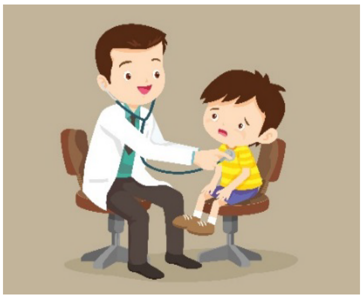

The doctor or nurse will do a checkup and some tests.

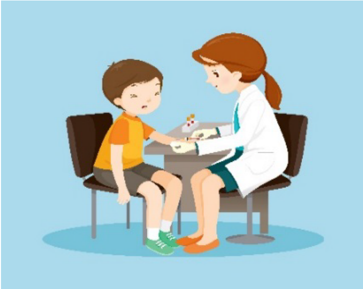

The doctor or nurse will take a little bit of your blood.

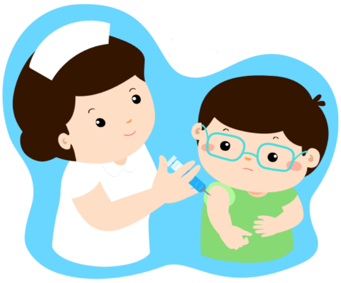

The doctor, a nurse, or your mom, dad, or the person who takes care of you will give you the study drug.

Will anything about the study upset you?

When the nurse or doctor takes some blood from your arm, you may feel a pinch. None of the other tests will hurt you, but you may feel some discomfort. Your mom, dad, or the person that cares for you will tell you about the tests before they are done.

When the doctor, nurse, or the person who takes care of you gives you the study drug, you may feel a pinch. The study drug may make you feel better or you may feel the same or worse. Because we are still studying this drug, we don't know everything about it.

Will doing the study help you?

We cannot promise that doing the study will help you. The study will tell us more about how people feel when taking the study drug for a long time. The study might also tell us if the study drug helps people with PH. We may learn things during this study that will help us understand more about the study drug. The study may also help us learn if we can help other children and grown-ups with PH.

ASSENT

Please ask the child (or, if unable, parent/guardian on his or her behalf) to circle all that he or she agrees with:

Have you read (or has your mom, dad, or the person that cares for you read) about doing the study?	✓ YES	✗ NO
Has somebody explained the study to you?	✓ YES	✗ NO
Do you understand what the study is about?	✓ YES	✗ NO
Do you understand that you do not have to do the study if you do not want to?	✓ YES	✗ NO
Have you asked all the questions you want?	✓ YES	✗ NO
Have you had your questions answered?	✓ YES	✗ NO
Do you understand it is OK to stop doing the study at any time?	✓ YES	✗ NO
Do you understand you can always ask more questions about the study, at any time, after today?	✓ YES	✗ NO
Do you want to do the study?	✓ YES	✗ NO

If any answers are "NO" or you **do not** want to do the study, **do not** write your name!

If you **do** want to do the study, please write your name and today's date. Your mom, dad, or the person who takes care of you must also want you to do the study.

Your name ____

Date____

DOCUMENTATION OF ASSENT

The research study and assent form was explained to:

____

Name of participant

Person Obtaining Assent

By signing below, I confirm that I have personally discussed this study with the participant and the procedures have been explained in terms he or she could understand, and that he or she has freely assented to participate in this study.

______________________________	_______________
Signature of person obtaining assent	Date
______________________________	
Printed name of person obtaining assent	
______________________________	_______________
Signature of parent/guardian	Date
______________________________	
Printed name of parent/guardian	
______________________________	_______________
Signature of second parent/guardian (if applicable)	Date
______________________________	
Printed name of second parent/guardian (if applicable)	

Assent Form for Adolescent Participants

Recommended for age 12 years

Study Title: **A Phase 2 Open-Label Multicenter Study to Evaluate the Efficacy, Safety, and Pharmacokinetics of Nedosiran in Pediatric Patients from Birth to 11 Years of Age with Primary Hyperoxaluria and Relatively Intact Renal Function**

**Principal Investigator:**	Investigator Name	Telephone: (xxx) xxx-xxxx
**Emergency Contact:**	Name	Telephone: (xxx) xxx-xxxx
**Available 24 hours a day:**	*[Should be a phone number accessible 24 hours (i.e., pager, cell) and answered by someone knowledgeable about the study]*	

Should the assenting adolescent decide not to take part in this study, the parent/guardian may not force the adolescent to take part.

We are asking you to be in a research study. Research is a way to test new ideas. Research helps us learn new things. A research study is a collection of tests and questions that helps us learn if a new drug works or not, and if it is safe to give to people.

In this study, we want to test a new drug. "Drug" is a name for a chemical product that has an effect in the body. Some drugs help the body. If a research study proves that a new drug helps the body, the drug can become a medicine.

By asking you, and others like you, to help with our study, we can find answers to some of the questions we have about children with primary hyperoxaluria (PH) «type 1, 2, or 3/type 1 or type 2», and if the new drug helps you and is safe.

You can ask your study doctor to explain anything in this form or about the study that you do not understand. You can ask as many questions as you like any time that you think of them. You can also talk about the study with your family and friends if you want to. Please take your time deciding whether you want to do the study or not.

This form gives you important information about the study. It will tell you why we are doing the study, and whether something good or something bad could happen to you during the study. If there is anything in this form that you do not understand, please ask questions.

You do not have to do the study if you do not want to. If you decide to do the study, you can stop doing it any time. No one will be upset with you if you decide to stop doing the study.

Why are you being asked to do the study?

You are being asked to take part in this study because you have PH. A new drug called nedosiran is being tested to see if it helps people with PH.

Your study doctor will tell you and your parent/guardian about this study, why it is being done, and what doing the study would mean to you. We would like to talk about the study with you and your parent/guardian so you can decide if you want to do the study. Whatever you decide is okay.

What is the purpose of this study?

The reason we are doing this study is to learn more about the study drug (nedosiran), like whether it is safe to give to people, and if it helps the livers of children with PH to make less oxalate and reduce symptoms of PH. The study drug is new and is not approved by any health authorities.

How many people will be in this study?

Up to 25 children from all over the world will be in this study.

Do you have to do the study?

You can say Yes or No. Either way is okay. Your study doctor or your parent/guardian cannot make you do the study.

If you agree to be in this study, your parent/guardian will also be asked to allow you to be in it. You and your parent/guardian will need to sign your names on this form. You will get a copy of this form to take home with you today.

If you decide to do the study but later change your mind, you can stop any time. Just tell your study doctor or your parent/guardian. If you decide to stop the study you may be asked to have some more tests to make sure you are okay, but then you will not do the study any more after those tests.

What do you have to do if you do the study?

If you want to do the study, you will have 11 appointments over the 7 months of the study. Two of these appointments will be telephone or video calls. At least 5 appointments will be a visit to the study site. Up to 4 appointments can be done at home, and a nurse will visit you to carry out study tests and procedures. Your appointments will be around every 2 weeks for the first 2 months of the study, then every month for the next 4 months. You may need to come back a few extra times if your study doctor feels it is better for you.

If you decide to do the study, you will be given the active study drug once a month during the study. The study doctor or nurse will give you your first 2 doses of the study drug through an

injection under the skin into your thigh or stomach area. After these 2 doses, if the study doctor agrees, you or your parent/guardian can give you the study drug every month via an injection into your thigh or stomach area – either at the study site in front of study staff, or at home in front of the visiting nurse.

Some of the tests and activities in the study will be repeated many times.

The study involves the following tests and activities:

Screening visit

The first visit (called the "Screening visit") is done just once. It will include all tests and activities described in this form, except getting the study drug. The Screening visit will allow the study staff to decide whether you are right for this study.

Study drug

You will receive the study drug once a month during the study. The study drug will be injected under the skin into your thigh or stomach area.

Interviews

A study staff member may ask you and your parent/guardian about your medical history, family background (including race and ethnicity), and any medicines you are taking. At every visit, the study doctor or nurse will ask you how you have been feeling while you've been in this study, and if you've had any kidney stones. You will be asked questions about how PH affects your life. You will also be asked about how the study visits and tests affect your life.

Physical examination

Examinations will be done at every on-site visit during this study. These examinations may include weight, height, blood pressure, heart rate, and breathing rate.

Scans and tests

An electrocardiogram, also called an ECG, is a test that looks at the electrical activity of your heart. The ECG pads may cause some redness and itching.

An echocardiogram (ultrasound of the heart) is a test that lets the doctor see how your heart moves as it pumps blood. You may feel some pressure when the wand is pressed against your chest area. At some visits, the study doctor may decide that this scan is not needed.

A kidney ultrasound will be done 2 times during the study. This will show an image of the size and shape of your kidneys, how the blood is flowing through your kidneys, and whether there are any stones or deposits in your kidneys. You may feel some pressure when the wand is pressed against your kidney area.

Blood collection

Every month during the study, we will collect blood samples from you. We will try not to prick you with the needle more than once each time. The amount of blood collected will not be more than the maximum that health authorities recommend for your weight. For example, for someone who weighs 30 kg, we will never collect more than about 24 mL (around 1.5 tablespoons) of blood at any one time, or 72 mL (about 5 tablespoons) in a 28-day period.

Taking blood may cause some pain, bleeding, or bruising at the spot where the needle is inserted into your skin. Sometimes, but not often, taking blood may cause fainting or infection.

Genetic test for PH

You will need to have a genetic test for PH, if this has not already been done. Study staff will either take some blood for this test, which is described above, or use a swab on the inside of your cheek. The cheek swab will not hurt you.

Urine collection

If you are able, you will be asked to pee into a cup at home and at the study site. You can do this in a bathroom without anyone watching you. The test is similar to those performed as part of regular medical care. Once a month throughout the study before the study appointment (7 times overall), you will need to collect several urine (pee) samples at home over 2 or 3 days (called spot urine samples). Your parent/guardian will have the details for these spot urine samples.

Recording fluid intake

You will be asked to keep track of how much fluid you drink each day during in the week before spot urine collections.

Pregnancy tests

If you are pregnant or nursing, you will not be allowed to participate in this study. If you are a girl and have already started having periods, you will be asked to take a urine pregnancy test before starting this study, and then every month while you are in the study. If you are able, you will be asked to pee into a cup at the study site for the pregnancy test, and you will be given pregnancy tests to take at home on the months you don't visit the study site.

The results of the pregnancy test will be shared with you and not with your parent/guardian. We strongly encourage you to share the results with your parent/guardian. If you are found to be pregnant, you will not be able to continue to be in this study and thus there is a risk that your parent/guardian would find out. If the pregnancy test is taken at home, your parent/guardian may need to assist you with taking the test and submitting the results to your study doctor. In this case, there is a risk your parent/guardian will see the pregnancy test results. If the urine pregnancy test is positive, please do not take your dose of nedosiran, and contact your study doctor or visiting nurse to have a pregnancy blood test to confirm the result.

Pregnancy risks

It is not known whether treatment with nedosiran may cause injury or harm to an unborn child if taken during pregnancy. For this reason, you should not become pregnant or get your partner pregnant while taking the study drug, nedosiran, and anyone who is pregnant is not allowed to take part in this study.

Birth control

If you have periods: You will need to take safety measures to prevent pregnancy while you are in this study. You can prevent pregnancy by not having sex or by using a form of birth control (for 4 weeks before you start taking the study drug, during the study, and for at least 12 weeks after your last dose of the study drug). Your study doctor will tell you which birth control is okay to use in this study. If you have questions about how to avoid pregnancy, talk to your study doctor, and they will provide you with information on choices of birth control or how to avoid getting pregnant. If you become pregnant during this study you should stop taking the study drug and contact your study doctor right away.

If you are a partner of someone who has periods: You should not get your partner pregnant during this study and for at least 12 weeks after your last dose of the study drug. You need to make sure you do things to avoid getting a partner pregnant. You can avoid pregnancy either by not having sex or by using birth control. If you have questions about how to prevent pregnancy, talk to your study doctor, and they will provide you with information on birth control choices.

Visit schedule

**Study Day number**	**Screening (up to 35 days before Day 1)**	**Day 1**	**Day 2**	**Day 15**	**Day 30**	**Day 45**	**Day 60**	**Day 90**	**Day 120**	**Day 150**	**Day 180 (end of study)**
**Visit window (days)**					**+/- 2**		**+/- 3**	**+/- 3**	**+/- 5**	**+/- 5**	**+/- 5**
**Visits that must be done at the clinic**	●	●			●			●			●
**Visits that may be done at home**			●				●		●	●	
**Telephone appointment**				●		●					
Sign informed consent	●										
Receive study drug		●			●		●	●	●	●	
Physical examination	●	●	●		●		●	●	●	●	●
Vital signs	●	●	●		●		●	●	●	●	●

Study Day number	Screening (up to 35 days before Day 1)	Day 1	Day 2	Day 15	Day 30	Day 45	Day 60	Day 90	Day 120	Day 150	Day 180 (end of study)
**Visit window (days)**					+/- 2		+/- 3	+/- 3	+/- 5	+/- 5	+/- 5
**Visits that must be done at the clinic**	●	●			●			●			●
**Visits that may be done at home**			●				●		●	●	
**Telephone appointment**				●		●					
Kidney ultrasound	●										●
Electrocardiogram	●	●			●			●			●
Echocardiogram (if possible)	●										●
PH genotyping	●										
Blood tests	●	●	●		●		●	●	●	●	●
Urine tests	●	●			●		●	●	●	●	●
Pregnancy test (if applicable)	●	●			●		●	●	●	●	●
Spot urine sample collection	●				●		●	●	●	●	●
Record fluid intake	●				●		●	●	●	●	●
Record kidney stone events		●	●		●		●	●	●	●	●
Questionnaires	●							●			●
Answer questions about how taking part in this study affects your daily life		●			●		●	●	●	●	●
Answer questions about how you have been feeling and any medicines you have taken	●	●	●	●	●	●	●	●	●	●	●

What happens after the study is over or if you decide to stop your participation?

After this study ends, you will be offered the option of joining another longer-term study with the same study drug, nedosiran.

If you choose to stop taking the study drug at any point during the study, or your study doctor or the study sponsor decides you have to stop the study treatment for any of the reasons mentioned in this form, you will be asked to return for additional follow-up visits. Attending these follow-up visits is very important, both for your safety and for learning more about the study drug.

Could you be hurt by doing the study?

Being in a research study involves inconveniences and risks. If you have any questions about any of the following possible risks, you should talk to your study doctor. The study drug may make you feel better or you may feel the same or worse. Because we are still studying this drug, we don't know everything about it. There may be other risks than the ones described below. If you feel unwell or have any new symptoms, please tell your parents/guardians and your study doctor or study staff.

The main risks of nedosiran are:

- Changes in blood tests that show that your liver is working differently than normal
- You may feel general muscle pain or weakness; please inform the study doctor right away if you feel this
- The place where the study drug was injected might have inflammation, mild reddening, soreness, itching, or swelling (called injection site reactions)

Will doing the study help you?

Doing the study may help with your PH by lowering the oxalate levels in your blood, but we don't know that for sure and we can't promise that you will receive any benefit by being in this study. Other studies have suggested that nedosiran could be more effective in people with PH1 than PH2 or PH3. We may learn things during this research study that will help us learn more about the study drug and if it can help people with PH.

What if something goes wrong during the study?

Your parent/guardian will be given a form that will tell them what to do if there is a problem and whom to call. If they have any questions or worries during the study, the study doctor and study staff want them to call the phone number on the first page of this form.

Will anyone else know you are doing the study? Will your medical details be kept private if you choose to be in this study?

If you decide to do the study, we will keep private the things we learn about you. We will use a code number instead of your name when your study doctor or nurse makes notes about how you are doing in the study. This coded information is what is shared with the company running the study.

The study doctor, study nurses, and some special employees of the companies that are working with your study doctor, will look at your full information to make sure the study is being done right. This is to protect you and any others who take the study drug.

During the study, we will collect information that is considered to be personal data by data protection laws. This includes information about your health, including testing your genes, and your gender and race. We collect this information so that we can keep accurate records of your reaction to the study drug. This is important so that we know whether the study drug will treat other people in future.

Your parent/legal guardian has consented on your behalf to do the study and they were told how your information is going to be used. If you have concerns about how we might use your information, you can ask questions now.

If you would like to know what information we have collected about you, or if you want to change or remove any information from your medical or study records, you and your parent/legal guardian can talk to the study doctor.

Who decides if you will do the study or not?

It is up to you and your parent/guardian. Both you and your parent/guardian need to agree for you to be part of the study. Your parent/guardian cannot make you do the study. If you would like to do the study, you must write your name on or sign this form. You will get a copy of this form to keep. When you think about whether you would like to do the study, please remember that you will need to follow the study doctor's instructions, go to all study appointments, and be given the study drug as the study doctor tells you to.

What is expected of you if you choose to do the study?

You will be asked to come to all visits and participate in all study procedures if you choose to be in the study. You will need to follow the study doctor's instructions, keep all study appointments, and receive the study drug as directed. Please talk with your parent/guardian and study doctor about the amount of time doing this study will take from you. Please think about how the study visits and being in the study might affect your life.

Can you stop doing the study before it is over?

Yes, you can stop doing the study at any time. You don’t have to give a reason. No one will be upset, and you won’t be punished in any way.

Can the study doctor make you stop doing the study?

The study doctor may make you stop doing the study if:

- You get sicker
- The study is stopped
- The study drug is no longer available
- You don’t qualify for the study anymore
- The doctor gets any new information that shows it is not a good idea for you to be in this study anymore

What choices do you have other than doing the study?

If you decide that you don’t want to do the study, here are some other choices you have:

- You may do another study, if one is available
- You may take your regular medicines and see your own doctor without having to do the study. Depending on the variant of PH you have, there may be an approved treatment your doctor can recommend
- You may talk about other choices with your own doctor and your parent/guardian

What happens if new information about the study drug is learned?

Sometimes during research, new things are found out about the study drug. Your study doctor will tell you about it if this happens.

ASSENT

It is okay to ask questions about what you have read or what someone has read to you.

You can circle things on this form that you want to know more about.

If you do not understand something, just ask so your study doctor or nurse or your parent/guardian can explain it.

If you do not want to do the study, do not write your name or sign this form.

If you do want to do the study, please sign your name and write today's date below.

☐ NO, I do not want to do the study

☐ YES, I want to do the study

☐ I understand that I can stop doing the study any time.

☐ I agree to inform my family doctor that I am taking part in this clinical study.

If you agree to be in the study, sign here: ______________________________________

Printed name of adolescent: ______________________________

Date: ______________________________

DOCUMENTATION OF ASSENT

The research study and assent form were explained to:

____
Name of participant

Person Obtaining Assent

By signing below, I confirm that I have personally discussed this study with the participant and the procedures have been explained in terms he or she could understand, and that he or she has freely assented to participate in this study.

______________________________	______________________________
Signature of person obtaining assent	Date
______________________________	
Printed name of person obtaining assent	
______________________________	______________________________
Signature of parent/guardian	Date
______________________________	
Printed name of parent/guardian	
______________________________	______________________________
Signature of second parent/guardian (if applicable)	Date
______________________________	
Printed name of second parent/guardian (if applicable)	

PARENTAL INFORMATION

For participation in the clinical study:

A Phase 2 Open-Label Multicenter Study to Evaluate the Efficacy, Safety, and Pharmacokinetics of Nedosiran in Pediatric Patients from Birth to 11 Years of Age with Primary Hyperoxaluria and Relatively Intact Renal Function

1 What is the purpose of this form?

Your child is being asked to take part in a clinical study. Clinical studies are needed to learn more about new drugs, such as how well they work and how safe they are. Global laws require that all new drugs being made are tested in clinical studies that will provide more understanding of how the new drug works in patients and affects a disease.

Taking part in this study is voluntary. Whether you allow your child to take part or not take part will in no way affect his or her current or future medical care.

Please read this information carefully. If you agree for your child to take part in this study, you will need to sign the attached consent form so that your doctor knows you agree for your child to participate in the study. The study doctor and/or a member of the study staff can answer any questions you may have and provide explanations. You will be asked to sign the attached consent form. If you would like your child to take part in this study, please do not sign the consent form until you understand the goals of the study and the procedures involved, and you have been informed of your and your child's rights.

If you decide to have your child take part in the study, a signed and dated copy of this Parental Information Form and the Parental Consent Form will be given to you.

If your child is old enough to assent: Your child does not have to take part in this study if he or she does not want to. For your child to participate, both you and your child will need to agree to take part. The study doctor and/or a member of the study staff can answer any questions your child may have. If your child decides to participate but later changes their mind and no longer wants to participate, you may stop their participation at any time, for any reason.

Your child will be asked to sign the attached assent form, indicating his or her expressed agreement to take part in the study. Please make sure your child only signs the form once they understand what the study involves. You will be given a copy of the signed Assent Form.

2 Why is this study being done?

This study is being run to test a new drug for use in people with primary hyperoxaluria (PH). The study drug is called nedosiran and is administered using a needle that is placed under the skin (subcutaneous injection). The purpose of this study is to learn more about how nedosiran works in the body and how well it helps the liver make less oxalate to reduce symptoms of PH in neonates, infants, and children (aged 11 years and under) with «primary hyperoxaluria type 1 (PH1), primary hyperoxaluria type 2 (PH2), or primary hyperoxaluria type 3 (PH3) / primary hyperoxaluria type 1 (PH1) or primary hyperoxaluria type 2 (PH2)».

The study drug, nedosiran, has not yet been approved by any Competent Authority (the people responsible for authorizing drug approvals, such as <Competent Authority for study site>), in the world and is an experimental treatment. We hope to learn more during this study about the safety of nedosiran and how it might work.

3 What is the drug that is being tested?

The study drug, nedosiran, is thought to work by reducing the amount of a key enzyme called lactate dehydrogenase A, which is involved in the production of oxalate. Enzymes are responsible for speeding up biological reactions. Reducing the activity and/or the amount of this enzyme means the liver may produce less oxalate.

Lowering the levels of oxalate could have a positive effect on patients with PH because most of the symptoms from PH are due to the excessive amounts of oxalate accumulating in the body. This could improve the quality and length of lives of people with PH.

4 How will the study be conducted?

In this study, your child will receive active study drug (nedosiran) every month for 6 months. All participants will be given the same study drug. About 25 participants will take part in this clinical study from 10 countries worldwide.

Your child will be administered nedosiran once a month by injection. For the first 2 months of the study, the study staff will administer nedosiran to your child once a month at the study site. After your child has been in the study for 2 months, this injection can be done at home, unless a visit to the study site is required. A nurse will be available to visit your home to help with home administrations and conduct the study assessments. If your child is 6 years of age or older and the study doctor agrees, it may be possible for you to administer the injection. The study staff will give you training on how to inject the study drug and provide you with detailed instructions. You will then be able to administer the injection at the study site and at home, while the visiting nurse is conducting the visit.

Unless you and your child cannot come to the study site, all other study procedures will be conducted at the study site by an investigator (study doctor) and/or their qualified study staff.

The sponsor of this study is Dicerna Pharmaceuticals, Inc. Dicerna is the company that is working to develop nedosiran, provides all funding for the work of this study, and is responsible for it. The principal investigator, who is the person in charge of ensuring the ethical conduct of this clinical study, is <enter Principal Investigator name, per the protocol>. The study doctor at each study site is being paid by the sponsor for their work in this study.

5 Why has my child been asked to take part in this study?

Your child is being asked to take part in this study because he or she has been diagnosed with «PH1, PH2, or PH3 / PH1 or PH2», and urine tests suggest that his or her kidneys function close to normal range for their age.

6 What will happen during this study?

Participating in a clinical study is a big commitment that will require your and your child's time with visits to the clinic and undergoing routine procedures that are designed to help determine whether or not nedosiran is a safe and effective treatment for PH. It is important to carefully read and understand the study requirements to determine if your child will be able to comply.

Your child will be in the study for around 7 months, including a screening process which can last up to 35 days. If you decide that you want your child to participate in this study, your child agrees (if they are old enough to assent), and he or she is eligible, he or she will start treatment with the study drug. You and your child will have visits at least once every 30 days. Several

urine collections will also need to be taken at home during this study – these urine collections are very important for the study.

Below is a list of important information pertaining to your child's participation in this study.

- **Notification to treating physicians:** You should inform your child's regular doctor or health care provider that your child is taking part in this study. You should also tell the study staff about any new treatment prescribed for your child by his or her regular doctor or health care provider while he or she is participating in this study.
- **Lifestyle considerations:** There are some things that you and your child may need to do differently while being in this study. These changes may be inconvenient.
    - Your child should not take vitamin C supplements, including multivitamins, for 24 hours before and during the collection of spot urine specimens.
    - Your child should avoid eating oxalate-rich foods, such as spinach, bran flakes, rhubarb, beets, potato chips, chocolate, and other foods. The study staff can provide information concerning which other foods contain high levels of oxalate.
    - Your child should continue the standard-of-care safety measures for those with PH including keeping high fluid intake levels, oral potassium supplement use, and treatment with vitamin B6 (if your child has PH1). Your child's intake of vitamin B6 should remain constant throughout the study if he or she has PH1.
    - You and your child will also need to perform spot urine collections at home over a 2- or 3-day period accurately and carefully; this may involve some inconvenience. These collections are very important for this study. The result will help determine if the study drug is helping your child.
- **Other clinical studies:** Taking part in other clinical studies during this study is not allowed.
- **Other medications:** Your study doctor will talk with you about any current medications your child is taking and any changes that may be required to participate in this study. Your child should not be given any vaccinations in the 7 days before or after study drug administration.
- **Pregnancy:** If your child has reached puberty, or reaches puberty during the study, it is very important for your child (or their partner) to avoid becoming pregnant during the study.
- **Screening process:** Prior to the start of the screening process, the study and its purpose will be explained to you. There will be time for you to ask questions. During this visit, you will first provide your consent for your child's participation in the study. The screening process will then start with genetic testing to confirm that your child has PH. This test will use a small blood sample, or, if your child weighs 11 kg or less, a swab will be taken from the inside of your child's cheek. If this has already been done as part of your child's regular medical care, the study doctor will use those results; the test will not need to be done again for this study. If the genetic testing confirms your child has PH, you and your child will return to the study site to complete all required screening assessments within 35 days of your consent.

These are the procedures and tests that your child will have during the study. Some will be done frequently and some will be done only a few times:

- **Injection of nedosiran:** The study doctor or a nurse will administer nedosiran to your child by injecting it under the skin (subcutaneous injection). After your child has been in the study for 2 months, it may be possible for you to administer this injection, if the study doctor agrees. The study staff will give you training on how to do this. Your child may find the injection painful or uncomfortable. More information about the risks of this injection are given further below in this form (**Section 8**, "What are the risks of taking part in this study?").

- **Blood draws:** Blood samples will be taken for several blood tests. Some of these tests will only be done once, while others may be repeated.

  The amount of blood collected will not exceed that specified by the European Commission. Depending on how much your child weighs, there is a maximum volume of blood that can be collected at a single time, and a maximum volume of blood that can be collected over a 28-day period. Study staff will never go over these maximum allowed volumes when collecting blood samples from your child.

  As an example, if your child weighs 12 kg, study staff will never collect more than 9.6 mL (less than 1 tablespoon) of blood from your child in one visit, or 28.8 mL (around 2 tablespoons) over a 28-day period.

  If the study staff find it difficult to collect blood from your child, they will not make more than 3 attempts to collect blood at any one time. It is possible that your child may feel some discomfort when the blood samples are being taken.

  If results from blood tests show that your child's liver function has changed since the start of the study, your child will be asked to give extra blood samples regularly (2 or 3 times a week) so we can monitor your child's liver function. If it is difficult for you and your child to get to the clinic, these tests can be done in a location closer to you.

- **Spot urine samples:** During the screening process, you will need to collect 6 urine samples (3 of which must be from the second urination of the morning) over 3 days. You will need to collect 4 on-treatment urine samples (2 of which must be from your child's second urination of the morning) over a 2-day period every month. You will be given detailed instructions for this. This is a very important test in the study. Your child will be asked not to take vitamin C (including multivitamins) for 24 hours before and during these spot urine sample collections.

- **Demographic details:** You will be asked your child's age, sex, ethnicity, and race, to help the study doctors learn if your child's background influences the way the study treatment affects his or her PH. Race needs to be collected to calculate estimated glomerular filtration rate (eGFR), a test of kidney function.

- **Physical examination:** Your child will be given a physical examination that will include checking the injection site and a review of body systems, and may include height (or length) and weight.

- **Vital signs:** Your child's blood pressure, pulse/heart rate, respiration rate, and body temperature will be measured. Your child may feel some pressure as the blood pressure cuff is inflated.

- **Medical history:** A medical history of your child's PH as well as any other illnesses your child may have experienced or is experiencing will be taken. You will be asked about previous or current medications your child is taking, including vitamins. During

the study, if your child experiences any kidney stone events, you will be asked to give details on these.

- **12-lead electrocardiogram (ECG):** Sticky patches attached to electrodes will be attached to your child's back, chest, arms, and legs. These electrodes will measure the electrical activity of your child's heart. This will be done in a few minutes and should not cause discomfort. The ECG sticky pads may cause some irritation and itchiness.
- **Echocardiogram:** This is an imaging of your child's heart, its shape, structure, and function, using sound waves. It is non-invasive. Your child may feel some discomfort from the wand that is pressed against his or her chest. If your child is upset with the examination and the study doctor can't get a clear image, they can decide to skip the echocardiogram without affecting the rest of the study. The echocardiogram will only be done if your child doesn't need any sedatives for it.
- **Kidney ultrasound:** This is a way of imaging your child's kidneys, bladder, and the tubes that connect them (ureters) through the use of sound waves. It is non-invasive. Your child may feel some discomfort from the wand that is pressed against his or her lower back. It will show if there are any kidney stones or other calcium deposits in your child's urinary system.
- **Pediatric burden assessment:** This assessment will help the investigator measure how much of a burden your child's taking part in this study is for you.
- **Questionnaires:** You will be asked to respond to some questionnaires at some visits. If your child is old enough and able to, they may also be asked to answer some questions. This will help the study doctor and staff understand how having PH affects your child's daily life.
- **Recording of fluid intake:** If your child is not currently breastfed, you will be asked to record how much fluid he or she drinks each day for at least 4 days before the spot urine collections.
- **Urinalysis:** This will be a typical urine test at the study site, separate from the spot collections. These tests will tell the investigator if there is anything abnormal in your child's urine, for example, blood or protein, and the pH (acidity) of your child's urine.
- **Pregnancy test:** If your child is of child-bearing potential (meaning that they have started having periods), they will be given urine pregnancy tests each month they are in the study.

While your child is in this study, your child will have at least 11 visits over the 6 months of the study, including 2 telephone or video calls. You and your child will be expected to make at least 5 visits to the study site, and the remaining 4 visits may be done at your home (or an agreed location) by a qualified and experienced research nurse (visiting nurse). The visiting nurse will conduct study procedures and assist with the urine collections, if necessary. During the scheduled telephone or video calls with the study doctor, you will be asked some questions about how your child is doing.

With your approval, the study site will send a registration form containing your contact details to a dedicated team at MDGroup, the company providing the research nursing services away from the study site. After this, the following steps will take place:

- The visiting nurse will call you and answer any questions you and your child may have about the service.

- The visiting nurse will arrange the time and date of your child's home (or agreed location) visits. They will phone to remind you of this a few days before each visit.
- On the day of your child's visit the visiting nurse will arrive at your home (or agreed location) at the pre-arranged time and start the visit.
- The visiting nurse will ask you and your child some questions, perform study assessments when needed, such as blood tests, physical examinations, and record your child's vital signs, and then either watch while you administer the injection of study drug or administer the injection themselves.

If the study drug is to be administered at home, please make sure it is kept in a regular refrigerator (between 2°C to 8°C, or 35°F to 46°F). You should not let the study drug freeze, or put it next to the freezer compartment or a freezer pack.

Home visits are optional. You and your child can choose to visit the study site in person for all of your child's visits. However, if you and your child are unable to visit the study site and do not want to have a nurse visit your home, you may not be able to participate in the study. If you or your child have any questions about a nurse visiting your child at home or at another location, the visiting nurse and the study site will work together to answer them.

The visit schedule below shows the visits that the procedures and tests described above will take place, as well as the window allowed for each visit (a visit window of +/- 2 days means the visit can take place up to 2 days before or after the date it is due to take place).

Visit schedule

**Study Day number**	**Screening (up to 35 days before Day 1)**	**Day 1**	**Day 2**	**Day 15**	**Day 30**	**Day 45**	**Day 60**	**Day 90**	**Day 120**	**Day 150**	**Day 180 (end of study)**
**Visit window (days)**					**+/- 2**		**+/- 3**	**+/- 3**	**+/- 5**	**+/- 5**	**+/- 5**
**Visits that must be done at the clinic**	●	●			●			●			●
**Visits that may be done at home**			●				●		●	●	
**Telephone appointment**				●		●					
Sign informed consent	●										
Receive study drug		●			●		●	●	●	●	
Physical examination	●	●	●		●		●	●	●	●	●
Vital signs	●	●	●		●		●	●	●	●	●
Kidney ultrasound	●										●
Electrocardiogram	●	●			●			●			●

**Study Day number**	**Screening (up to 35 days before Day 1)**	**Day 1**	**Day 2**	**Day 15**	**Day 30**	**Day 45**	**Day 60**	**Day 90**	**Day 120**	**Day 150**	**Day 180 (end of study)**
**Visit window (days)**					**+/- 2**		**+/- 3**	**+/- 3**	**+/- 5**	**+/- 5**	**+/- 5**
**Visits that must be done at the clinic**	●	●			●			●			●
**Visits that may be done at home**			●				●		●	●	
**Telephone appointment**				●		●					
Echocardiogram (if possible)	●										●
PH genotyping*	●										
Blood tests	●	●	●		●		●	●	●	●	●
Urine tests	●	●			●		●	●	●	●	●
Pregnancy test**	●	●			●		●	●	●	●	●
Spot urine sample collection***	●				●		●	●	●	●	●
Record fluid intake	●				●		●	●	●	●	●
Record kidney stone events		●	●		●		●	●	●	●	●
Questionnaires	●							●			●
Answer questions about how taking part in this study affects your daily life		●			●		●	●	●	●	●
Answer questions about how your child has been feeling and any medicines your child has taken	●	●	●	●	●	●	●	●	●	●	●

* If your child weighs 11 kg or less, genotyping will be performed using a cheek swab. Children weighing over 11 kg will have genotyping performed by a blood sample.

** Pregnancy tests will be required only for people of child-bearing potential, and will be performed using urine samples. Positive urine tests will be confirmed using a blood sample.

*** During the screening period you will be asked to collect 6 spot urine samples (3 of these must be from the second urination of the morning) over a 3-day period. After treatment

begins, you will be asked to collect 4 spot urine samples (2 from the second urination of the morning) over a 2-day period before each visit specified.

7 What happens after the study is over or if I decide to stop my child's participation?

After this study ends, you and your child will be offered the option of joining another longer-term study with the same study drug, nedosiran.

If you or your child have chosen for your child to stop taking the study drug at any point, or your child's study doctor or the study sponsor have decided your child has to stop the study treatment (possible reasons for this are given below in **Section 17**), your child will be asked to return for an additional follow-up visit.

Attending this follow-up visit is very important, both for your child's safety and for learning more about the study drug your child has taken.

8 What are the risks of taking part in this study?

Some procedures may cause your child some pain or discomfort. For example, pain and/or bruising at the puncture site may occur during or after a blood draw. Swelling of a vein or in very rare cases, a blood clot, could also occur.

Nedosiran may present risks that are not well-known or understood. Therefore, there may be unforeseeable risks associated with participating in this research study. As of November 2021, around 71 healthy volunteers, 24 people with renal disease but not PH, and 60 people with PH, have been given either placebo or nedosiran in a clinical study.

In these studies, the most common side effects so far are injection site reactions, which is a response of the body to injuries, resulting in inflammation, mild reddening, soreness, itching, or swelling at the place where the study treatment was injected.

In studies with other drugs of the same class as nedosiran, in addition to injection site reactions, there have been events such as elevated liver enzymes which may be indicative of abnormal liver function. Other symptoms that your child may develop are fatigue, nausea, vomiting, abdominal pain or tenderness around the liver, fever, or rash. Your child may also have general muscle pain or weakness from the study drug. Observations from older drugs of the same class as nedosiran include changes in blood clotting, a reduction in blood platelets (called thrombocytopenia), activation of parts of the immune system (such as so called cytokines and complement factors), and mild or moderate abnormalities of the liver. As the study drug (nedosiran) used in this research may have risks that are not well-known or understood, there may be other risks that are not yet known. If your child has any new symptoms or unwanted effects, including general muscle pain or weakness, you should make a note of them and report them to the study doctor at your next scheduled visit. **Please inform the study doctor immediately at the telephone number listed in Section 19 (page 13) of any serious unwanted effects.**

In addition, there might be a risk of side effects, perhaps serious side effects, which we cannot predict or know about ahead of time, including effects on your child's liver, kidneys, heart, lungs, or other organs. In a very rare situation these side effects could be permanent or cause death.

Pregnancy risks

It is not known whether treatment with nedosiran may cause injury or harm to an unborn child if taken during pregnancy. For this reason, anyone who is pregnant is not allowed to take part in this study, and all participants and their partners should avoid becoming pregnant while the participant is taking nedosiran, and for 12 weeks after their last dose. Participants and their partners must agree to not have sexual intercourse, or must use a reliable, effective birth control during the study and for at least 12 weeks after the last dose of nedosiran. The study doctor will discuss the acceptable forms of birth control, if applicable.

If your child has started having periods, or starts during this study, they are considered of child-bearing potential. Urine pregnancy tests will be performed for participants of child-bearing potential before the research begins and will be done monthly throughout the study. A blood pregnancy test will be performed following a positive urine pregnancy test, whenever a menstrual cycle is missed, or when pregnancy is otherwise suspected.

The results of the pregnancy tests are confidential. Your child may need to take some pregnancy tests at home and report the results to your study doctor. If the urine pregnancy test is positive, or you or your child think they might be pregnant for any other reason, you or your child must contact the study doctor or visiting nurse to have a pregnancy blood test to confirm the result. If the urine pregnancy test is positive during a study visit, nedosiran should not be administered.

For study participants of child-bearing potential: If your child becomes pregnant during the study or within **12 weeks** of their last dose of nedosiran, please **inform the study doctor/study site right away (see Section 19, page 13)**. Your child's participation in the study will be stopped. Your child will need to remain in contact with the study doctor until **6 to 8 weeks** after the pregnancy ends (either through termination or birth). This is for safety reasons.

It is not known whether treatment with nedosiran could cause harm to a breastfeeding infant if passed through the breast milk. For this reason, anyone who is breastfeeding is not allowed to take part in this study.

It is not known whether treatment with nedosiran has any effect on eggs in ovaries. In the unlikely event your child may have eggs collected and frozen for the purpose of using them later in fertility treatment, this should not be done during the 4 weeks before they start treatment, while they are taking part in the study, and for at least 12 weeks after their last dose of nedosiran.

For study participants whose partners are of child-bearing potential: If your child is sexually active while taking part in this study, and has a partner who is of child-bearing potential, or already pregnant or breastfeeding, the study doctor will discuss the contraception they should use during the study and for at least **12 weeks** after treatment.

If your child's partner becomes pregnant during the study or within **12 weeks** of their last dose of DCR-PHXC, please **inform the study doctor immediately**. As the risk to their partner and the unborn baby is unknown, it is preferable for their partner to consult with a specialist during and after their pregnancy. The sponsor will also request their partner's consent to collect confidential information about their health and that of the baby for a period of **6 to 8 weeks** after the pregnancy has ended.

9 Does taking part in this study provide any benefit?

Taking part in this clinical study does not mean that your child will benefit from the treatment he or she is given. The purpose of this study is to learn more about the study drug, nedosiran, when it is given long term.

Treatment with nedosiran may or may not improve your child's PH symptoms. Study DCR-PHXC-201 showed that nedosiran lowered urinary oxalate in people who have PH1, but results were not consistent for people with PH2: some people's urinary oxalate decreased, some increased, and some stayed the same. Study DCR-PHXC-104 in people with PH3 showed that after one dose of nedosiran, urinary oxalate decreased some but not as much as researchers expected. Although your child may not receive any benefit themselves, their participation may provide new information about the treatment of PH.

10 What other treatments are available?

There is one treatment for PH1 which is approved in the United States and Europe, but no treatments available for PH2 or PH3. The study doctor can discuss any alternative treatments for your child with you.

In general, patients with PH must follow strict guidelines in respect to high water intake and minimizing their dietary intake of oxalate-rich foods. However, even with their best efforts, many people may progress to more serious renal disease in their mid-30s. This happens more in patients with PH1 than in patients with PH2 or PH3. In patients with more advanced disease, especially in patients with PH1, kidney and liver transplants are an option, but these present a number of complications, such as the body rejecting the donor organ.

11 What about confidentiality?

By signing this form, you agree to let the study doctor permit certain groups to review information about your child taking part in the study and your child's medical records. These might include the study sponsor, its designees, ethics committees/institutional review boards, and regulatory authorities. For more information on how your personal data is handled, please see the section titled, "Information Concerning Data Protection" at the end of this form.

All data and medical records tied to your child's taking part in this study will be kept private, except where required by law. If information about the study is published, it will be written in such a way that your child cannot be recognized. Your child will be identified by a unique study number and not by his or her name whenever possible.

Data collected about your child for the study may be included in reports. These reports will be submitted to authorities in various countries so that one day, the study drug may be available to all patients who have PH. Your child will not be identified in these reports.

During the study, a "monitor" may visit the study site. The monitor is responsible for making sure that the clinical study is being performed properly. The monitor will have access to your child's original medical records (including confidential data that identifies him or her by name). The monitor will compare these records to study forms for correctness.

Genetic examinations can never be fully anonymized and may produce indications of existing diseases (or of ones that will only occur in future), which are not connected with the objectives of the trial (incidental findings). The study doctor will inform you of such findings if you wish. However, the communication of incidental findings may have far-reaching, or even negative consequences for you and the lives of your blood relatives. For example, you may be obliged to give this information when concluding an insurance contract or in the context of a health examination.

12 What will happen to my child's study samples?

Study staff will collect blood, urine, and cheek swab (if applicable) samples from your child as described in this form. Samples will be kept by a central laboratory and destroyed after 5 years or per local regulations.

By signing this informed consent form, you allow the study staff to provide these samples to the sponsor and others working with the sponsor. Your child's blood samples will be used to test for safety, to measure immune response, how much of the study drug is present, and effects of the drug in your child's body. There may be some tests for your child's samples that have not yet been identified that we will need you and your child's permission to test in the future. If you or your child do not want to give permission for this testing, it will not affect how the study doctor will treat your child or his or her participation in this study.

13 Will it cost me anything for my child to be in this study?

There will be no cost to you or your child for taking part in this study. Your child will be provided with all study drugs, examinations, evaluations, and medical care related to the study at no cost to you or your child.

14 Will I be paid for my child's participation in this study?

You will be reimbursed for reasonable expenses associated with study visits, such as parking, transportation, and meals per local regulations. Any approved travel or medical companion will be reimbursed for their expenses associated with study visits.

This study will use a service from ClinEdge, www.clin-edge.com, to manage all payments associated with your child's participation in study visits, your time and travel related to participation in the study. Additionally, they will provide support as a travel agent and will book all your study related travel and accommodations. The sponsor has contracted with ClinEdge to provide these services, but the study site and ClinEdge are separate entities and have no other relationship. ClinEdge is solely responsible for the security of any information you provide to them.

We understand that by allowing your child to take part in this study you and your child are committing to adhering to the restrictions in **Section 6**, along with committing to visit the clinic on a number of occasions. This might mean you have to take days off work or travel long journeys to get to the clinic. For these reasons you will receive XXX per visit for participating in the study.

Reimbursement for loss of wages may be considered a source of taxable income in your country of residence. It is your responsibility to declare these payments in accordance with local tax regulations.

15 What happens if something goes wrong?

You will be asked to contact the study doctor if you feel that your child has been injured because of being in this study **(see Section 19, page 13)**.

There is a special study insurance that may help your child if he or she has an injury as a direct result of being in this study. If your child is injured, please tell the study doctor right away and then contact the insurance company immediately after. Otherwise, you and your child may lose insurance coverage. Please inform the insurance company directly or inform the study doctor **(see Section 19, page 13)**, who then will inform the insurance company on your child's behalf. In this case, the study doctor will provide you and your child with a copy of the report. The

insurance was taken out with «enter company, address, telephone no., fax no., and insurance no.».

A separate insurance policy is in place to cover your child in the case of an accident on the way to or from the study site. This insurance has been taken out with «insert company (address, telephone no., fax no.)». As with the research-related injury insurance above, it is important that you and your child inform «enter company» immediately in the case of an accident. Otherwise, your child may lose his or her insurance coverage.

16 Does my child have to take part in this study?

No; taking part in this study is voluntary. You and your child are free to refuse to take part or withdraw from the study at any time without penalty or loss of benefits to which your child is otherwise entitled. You and your child's decision to take part or not take part will in no way affect his or her current or future treatment. You will be given ample time and chance to ask questions about the details of this study and to decide if you want your child to take part.

Once the clinical study has started, if you or your child chooses to withdraw, your child will be asked to undergo a final examination, including spot urine samples prior to the visit. During this examination blood will be drawn and additional tests will be performed to monitor your child's safety. You and your child have the right to refuse to take part in further data collection or follow-up examinations after withdrawing from the study. If you or your child chooses to withdraw from the study, the sponsor can still use your child's information that they have already collected. Any unused blood or urine samples that were collected can be destroyed at your request. If you choose to have your child's samples destroyed please inform the study doctor.

17 Can my child be removed from this study without my permission?

The study doctor or the sponsor of this clinical study may decide to withdraw your child from the study without your consent or your child's assent. The reasons for withdrawal might be the following:

- If you or your child are unable to fulfill the requirements of the study
- If you or your child are not complying with study protocol
- If a clinical adverse event, laboratory abnormality, or other medical condition or situation occurs, so that it is not in your child's best interests to continue in the study
- If your child meets a study exclusion criterion that he or she has either newly developed or was not previously recognized
- If your child develops another serious illness
- If liver function studies indicate a new abnormality or your child develops symptoms of fatigue, nausea, vomiting, abdominal pain or tenderness around the liver, fever, rash, or other laboratory abnormalities that may affect the liver
- If the sponsor decides to end the study or your child's taking part in the study

18 What are my and my child's responsibilities as a participant in this study?

As a participant in this study, you and your child will have to do the following:

- Attend all visits as scheduled

- Provide honest answers to all questions asked by the study doctor and staff
- Follow the lifestyle considerations of study participation
- Follow the study doctor's instructions

The success of this study in providing new information for the treatment of PH depends on the cooperation of study participants. Therefore, you and your child will need to keep appointments and follow the instructions of your child's study doctor.

19 Who do I contact in case of an emergency?

The study doctor and address of your child's study site is:

Name of study doctor

Institution

Address

Telephone No.

Fax No.

For questions concerning this study or **in case of emergency**, please contact the study doctor at your child's study site. If you are not able to reach the study doctor at the telephone number listed here, please contact your child's family doctor. If it is a true emergency situation, visit the closest emergency department.

20 Who do I contact if my child or I have questions about this study?

Name	«enter IRB/IEC information»
Institution	
Address	
Telephone No.	
Fax No.	

21 Will my child and I be informed of new information about this study?

In the event that important new findings during the course of the research become available, which might be relevant to you or your child's willingness to continue in the study, this information will be provided to you and your child in a timely manner.

22 Who has reviewed this study?

This study will be conducted under the applicable regulatory requirements in your country and Good Clinical Practice rules. These rules are internationally accepted guidelines for performing studies that are safe and produce valid data. The necessary study documents will be submitted to the relevant institutional review boards/ethics committees. The study will begin only after approval has been received.

23 Study available on clinicaltrials.gov and clinicaltrialsregister.eu websites

A description of this clinical trial will be available on http://www.ClinicalTrials.gov, as required by United States (US) law. This Web site will not include information that can identify your child. At most, the Web site will include a summary of the results. You can search this Web site at any time.

A description of this clinical trial will be available on https://www.clinicaltrialsregister.eu, as required by European (EU) law. This Web site will not include information that can identify your child. At most, the Web site will contain a summary of the results. You can search this Web site at any time.

PARENTAL CONSENT FOR CHILD TO TAKE PART IN A RESEARCH STUDY

A Phase 2 Open-label Multicenter Study to Evaluate the Efficacy, Safety, and Pharmacokinetics of Nedosiran in Pediatric Patients from Birth to 11 Years of Age with Primary Hyperoxaluria and Relatively Intact Renal Function

My child's taking part in this clinical study is voluntary. My child has the right to stop taking part in the study at any time, without specifying our reasons, and without penalties or loss of benefits to which my child is otherwise entitled. I understand that it is in my child's best interest and for the safety of my child's health to have a final examination and to allow the collection of blood samples needed to perform laboratory tests for safety reasons. My child can also be excluded from continuing to take part in the study if the study doctor considers it necessary for my child's safety.

My child and I are ready to follow the instructions given by the study doctor and his or her staff who are conducting the study. My child and I have answered all questions presented to us to the best of our knowledge.

I confirm with my signature that I have had enough time to read and understand the information in this consent form. My questions have been answered to my satisfaction. My child and I have the right to ask further questions as they come up during the study. I have been informed about the existing study insurance policy.

My child and I have been told whom to contact if anything goes wrong in the study, if we have any questions, or my child is hurt in any way.

I agree to have my child take part in the clinical study on the basis of the information provided to us by the study doctor conducting the clinical study.

I have received a copy of these forms.

My child and I agree to the review of my child's data collected by the study doctor, by persons authorized and obligated to secrecy by the sponsor, the responsible national (and foreign) regulatory authorities, or the responsible federal authority as far as this is necessary for the review of the study. My child and I release the investigator of his or her obligation of medical confidentiality (privacy) to the extent that is required to permit such review.

If, during this study, my child is hospitalized or receives treatment at a health care facility other than the study site, my child and I agree to allow the study doctor access to medical records related to any treatment received and hospital-release papers.

☐ I agree to inform my child's family doctor of my child's taking part in this clinical study.

<u>Visiting nurses for remote visits:</u> Please check one of the boxes below. Remote visits are optional; you do not have to agree to register for research nursing services.

☐ I ***<u>AGREE</u>*** for the study site to register my contact details with MDGroup.

☐ I ***<u>DO NOT AGREE</u>*** to register for the visiting nurse service, and will travel to the study site for all the scheduled visits of this study. I understand that if we are unable to travel to the study site, my child may not be able to participate in the study.

Unspecified future testing of your child's stored blood and urine samples: Please check one of the boxes below. Your decision will not affect your child's ability to participate in the study. You will not be compensated for any use of your stored samples.

☐ I ***AGREE*** that my child's samples can be analyzed for future tests.

☐ I ***DO NOT AGREE*** that my child's samples can be analyzed for future tests.

Study participant no.: ____________________

Parent/guardian initials: ______________

____
Printed name of parent/guardian

____ ____
Signature of parent/guardian Date

Parent/guardian initials: ______________

____
Printed name of second parent/guardian (if applicable)

____ ____
Signature of second parent/guardian Date

____
Printed name of the person obtaining consent

____ ____
Signature of the person obtaining consent Date

For use in the UK, France, Belgium and Luxembourg, plus any EU country that does not specifically require consent for processing in a clinical trial.

INFORMATION CONCERNING DATA PROTECTION

For taking part in the research study:

A Phase 2 Open-label Multicenter Study to Evaluate the Efficacy, Safety, and Pharmacokinetics of Nedosiran in Pediatric Patients from Birth to 11 Years of Age with Primary Hyperoxaluria and Relatively Intact Renal Function

Information We Collect

During this clinical study, your child's personal data and medical findings will be collected for the purposes of scientific research. These data will be stored and reviewed according to the law.

For this study, Dicerna Pharmaceuticals, Inc. is the Data Controller with respect to the study data and Dicerna are the Data Controller with respect to your child's medical records. A Data Controller is responsible for deciding how your child's personal data are collected and used and will ensure that your child's personal data are processed in accordance with all applicable data protection laws, including the General Data Protection Regulation (GDPR) and «applicable local regulations», which are designed to protect your child's right to privacy.

The purpose of the processing of personal data related to your child's health condition is to achieve the study objectives as described above. As the sponsor of this study, Dicerna Pharmaceuticals, Inc. has a legitimate interest to process your child's personal data to conduct the clinical study. The benefits of the study will be to gain information about primary hyperoxaluria and the safety and effectiveness of nedosiran. Such processing is necessary for scientific research purposes.

Also, the sponsor has a legal obligation to process some parts of your child's data in order to ensure your child's safety and the integrity of the results of the study to develop new medicines. Such processing is necessary in the public interest in the field of public health e.g., ensuring high standards of quality and safety of health care and of medicinal products.

As the sponsor is not established in the European Union / UK, it has appointed Dr Phil Griffiths with a registered office at The DPO Ltd, Suite CDV 47286, 350 Chemin du Pre Neuf 38350 La Mure, France/The DPO Ltd, Capital Tower, Greyfriars Road, Cardiff CF10 3AZ, United Kingdom as their Data Protection Representative.

If you choose for your child to be in this study, the study doctor, on behalf of the sponsor, will gather and use your child's personal data to conduct the study. The personal data may include:

- Your child's name, address, and contact details
- Date of birth
- Demographic information, such as race and gender
- Medical history, including past and present medical records
- Information from your child's study visits, including test results, questionnaires, completed forms, images, and blood, urine, and genetic samples

How We Use the Information

The sponsor and those working with them may use the personal data sent to them:

- To see if the study drug works
- To see if the study drug is safe
- For other research activities related to the study drug
- To ensure that the study is being conducted properly and that applicable laws and procedures are being followed
- To make any reports required by applicable laws

Security

The study doctor and site staff will keep your child's personal data confidential and will not pass this information to sponsor in an identifiable form. Your child will be assigned a unique study identification number which will be their identifier in the clinical study documentation. Your child's number will never be reassigned or reused for any reason. The study doctor will be required to maintain a list linking the assigned study number with your child's name and the sponsor will never receive that list. The study doctor and site staff will use the information on this list as needed, to contact you about the research study, and make sure that relevant information about the study is recorded for your child's care, and to oversee the quality of the study.

When We Share this Information

The study doctor may share your child's non-identifiable personal data with:

- Dicerna Pharmaceuticals, Inc. or its designee (any company they use to oversee or conduct the study)
- Premier Research, the contract research organization designated by the sponsor to oversee this study
- «IRB/IEC/ IEC», which is a group of people who review the ethics of human research
- A safety review committee
- The «local regulatory authority»
- The Food and Drug Administration (FDA) and other US governmental agencies
- The Department of Health and Human Services (DHHS) «or local equivalent»
- Governmental agencies for other countries
- Other companies or agencies working with (or owned by) the sponsor
- Your child's family doctor, as necessary

Certain individuals from Dicerna Pharmaceuticals, Inc. and its representatives and regulatory organizations may look at (monitor) your child's medical and research records to check the accuracy of the research study and so will have access to your child's identifiable personal data. This may be done in person while on site. Your child's medical and research records may also be reviewed offsite via screen-share with site personnel or through a secure transmission of de-identified documents. This process is called "remote source document verification" and

will follow all applicable local regulations. Dicerna Pharmaceuticals, Inc. shall ensure that only secure processes are used to perform remote source document verification.

Dicerna Pharmaceuticals, Inc. will only receive information without any identifying data. The people who analyze the information will not be able to identify your child and will not be able to find out your child's name or contact details.

In addition, in order to provide nursing services away from the study site to your child, Dicerna Pharmaceuticals, Inc. has authorized a vendor, MDGroup, to process personal data that may identify your child. This identifiable data will not be passed on to the sponsor and the sponsor remains responsible for the security of your child's personal data. Furthermore, where it is necessary to send your child's samples to a laboratory or to deliver materials to your child directly at home, your child's name and address will be shared by the study doctor in order to provide instructions to the courier.

Future Research

When you agree for your child to take part in a research study, the information about your child's health and care may be provided to researchers conducting other research studies in this organization and in other organizations. These organizations may be universities, healthcare organizations or companies involved in health and care research. Your child's information will only be used by organizations and researchers to conduct research in accordance with applicable laws and current Good Clinical Practice regulations.

«EITHER:

This information will not identify your child and will not be combined with other information in a way that could identify your child. The information will only be used for the purpose of health and care research and cannot be used to contact your child or to affect their care. It will not be used to make decisions about future services available to your child, such as insurance.

OR:

Your child's information could be used for research in any aspect of health or care, and could be combined with information about them from other sources held by researchers, the health service or government.

Where this information could identify your child, the information will be held securely with strict arrangements about who can access the information. The information will only be used for the purpose of health and care research, or to contact you and your child about future opportunities to participate in research. It will not be used to make decisions about future services available to your child, such as insurance.

Where there is a risk that your child can be identified their data will only be used in research that has been independently reviewed by an ethics committee.»

International Transfers

Once your child's personal data has been shared with authorized users, it may no longer be protected by privacy laws in your country. Your child's data may be transferred to other countries for processing, including those that do not have data protection legislation as strong as in your country. The sponsor will ensure that all transfers are made in accordance with applicable laws and that there are appropriate security measures in place to protect your child's personal data.

For this clinical study, your child's data will be transferred to countries outside of the «UK / European Economic Area *(delete/amend as applicable)*» including «country/ies *(delete/amend*

as applicable)». The legal mechanism that will be used to transfer your child's personal data is Standard Contractual Clauses pursuant to the Commission Decision C (2010) 593, a copy of which is available to you upon request to the study doctor.

How Long We Will Keep the Information

Your child's personal data will be retained for «[x] years/a period determined by applicable laws and regulatory requirements, which could be for the entire life of the study drug or a longer period».

Your Child's Rights

We use your child's personal data to conduct research to improve health and care. As a pharmaceutical company Dicerna Pharmaceuticals, Inc. has a legitimate interest in using information relating to your child's health and care for research studies, when you agree that they can take part. This means that we will use your child's data, collected during this study, in the ways needed to conduct and analyze the research study. You / your child may request access to your child's personal data, for it to be corrected or deleted, or you may object to the processing of your child's personal data. However, your child's rights to access, change or move their information are limited, as we need to manage your child's information in specific ways, in order for the research to be reliable and accurate. If you withdraw your child from the study, we will keep the information about your child that we have already obtained. To safeguard your child's rights, we will use the minimum personally-identifiable information possible.

If you wish to raise a complaint on how we have handled your child's personal data, you can contact the study doctor in writing or the sponsor's Data Protection Officer by using this email: sar@thedpo.co.uk and they will investigate the matter. If you are not satisfied with the response or believe we are processing your child's personal data in a way that is not lawful you can complain to «country specific Data Protection Authority and their contact details/website».

For use in the US and ROW and, in particular, can be used in the EU if the above version is rejected/not allowable, e.g., Germany.

INFORMATION CONCERNING DATA PROTECTION

For taking part in the research study:

A Phase 2 Open-label Multicenter Study to Evaluate the Efficacy, Safety, and Pharmacokinetics of Nedosiran in Pediatric Patients from Birth to 11 Years of Age with Primary Hyperoxaluria and Relatively Intact Renal Function

Information We Collect

During this clinical study, your child's personal data and medical findings will be collected. These data will be stored and reviewed according to the law. This requires your consent prior to taking part in the study.

For this study, Dicerna Pharmaceuticals, Inc. is the Data Controller with respect to the study data and Dicerna are the Data Controller with respect to your child's medical records. A Data Controller is responsible for deciding how your child's personal data are collected and used and will ensure that your child's personal data are processed in accordance with all applicable data protection laws, including the General Data Protection Regulation (GDPR) and «applicable local regulations», which are designed to protect your child's right to privacy.

As the sponsor is not established in the European Union, it has appointed Dr Phil Griffiths with a registered office at The DPO Ltd, Suite CDV 47286, 350 Chemin du Pre Neuf 38350 La Mure, France as their Data Protection Representative.

If you choose for your child to be in this study, the study doctor, working with the sponsor, Dicerna Pharmaceuticals, Inc., will gather and use your child's personal data to conduct the study. The personal data may include:

- Your child's name, address and contact details
- Date of birth
- Demographic information, such as race and gender
- Medical history, including past and present medical records
- Information from your child's study visits, including test results, questionnaires, completed forms, images, and blood, urine, and genetic samples

How We Use the Information

The sponsor and those working with them may use the personal data sent to them:

- To see if the study drug works
- To see if the study drug is safe
- For other research activities related to the study drug
- To ensure that the study is being conducted properly and that applicable laws and procedures are being followed
- To make any reports required by applicable laws

Security

The study doctor and site staff will keep your child's personal data confidential and will not pass this information to sponsor in an identifiable form. Your child will be assigned a unique study identification number which will be their identifier in the clinical study documentation. Your child's number will never be reassigned or reused for any reason. The study doctor will be required to maintain a list linking the assigned study number with your child's name and the sponsor will never receive that list. The study doctor and site staff will use the information on this list as needed, to contact you about the research study, and make sure that relevant information about the study is recorded for your child's care, and to oversee the quality of the study.

When We Share the Information

The study doctor may share your child's non-identifiable personal data with:

- Dicerna Pharmaceuticals, Inc. or its designee (any company they use to oversee or conduct the study)
- Premier Research, the contract research organization designated by the sponsor to oversee this study
- «IRB/IEC/ IEC», which is a group of people who review the ethics of human research
- A safety review committee
- The «local regulatory authority»
- The Food and Drug Administration (FDA) and other US governmental agencies
- The Department of Health and Human Services (DHHS) «or local equivalent»
- Governmental agencies for other countries
- Other companies or agencies working with (or owned by) the sponsor
- Your child's family doctor, as necessary

Certain individuals from Dicerna Pharmaceuticals, Inc., its designees, and regulatory organizations may look at (monitor) your child's medical and research records to check the accuracy of the research study and so will have access to your child's identifiable personal data. This may be done in person while on site. Your child's medical and research records may also be reviewed offsite via screen-share with site personnel or through a secure transmission of de-identified documents. This process is called "remote source document verification" and will follow all applicable local regulations. Dicerna Pharmaceuticals, Inc. shall ensure that only secure processes are used to perform remote source document verification.

Dicerna Pharmaceuticals, Inc. will only receive information without any identifying data. The people who analyze the information will not be able to identify your child and will not be able to find out your child's name or contact details.

In addition, in order to provide nursing services away from the study site to your child, Dicerna Pharmaceuticals, Inc. has authorized a vendor, MDGroup, to process personal data that may identify your child. This identifiable data will not be passed on to the sponsor and the sponsor remains responsible for the security of your child's personal data. Furthermore, where it is necessary to send your child's samples to a laboratory or to deliver materials to your child directly at home, your child's name and address will be shared by the study doctor in order to provide instructions to the courier.

Future Research

If you agree, the information about your child's health and care may be provided to researchers conducting other research studies in this organization and in other organizations. These organizations may be universities, healthcare organizations or companies involved in health and care research. Your child's information will only be used by organizations and researchers to conduct research in accordance with applicable laws and current Good Clinical Practice regulations.

«EITHER:

This information will not identify your child and will not be combined with other information in a way that could identify them. The information will only be used for the purpose of health and care research and cannot be used to contact your child or to affect their care. It will not be used to make decisions about future services available to your child, such as insurance.

OR:

Your child's information could be used for research in any aspect of health or care, and could be combined with information about your child from other sources held by researchers, the health service or government.

Where this information could identify your child, the information will be held securely with strict arrangements about who can access the information. The information will only be used for the purpose of health and care research, or to contact you and your child about future opportunities to participate in research. It will not be used to make decisions about future services available to your child, such as insurance.

Where there is a risk that your child can be identified their data will only be used in research that has been independently reviewed by an ethics committee.»

International Transfers

Once your child's personal data has been shared with authorized users, it may no longer be protected by privacy laws in your country. Your child's data may be transferred to other countries for processing, including those that do not have data protection legislation as strong as in your country. The sponsor will ensure that all transfers are made in accordance with applicable laws and that there are appropriate security measures in place to protect your personal data.

Transfers Out Of The European Economic Area

For this clinical study, your child's data will be transferred to countries outside of the European Economic Area including «country/ies *(delete/amend as applicable)*». The legal mechanism that will be used to transfer your child's personal data is Standard Contractual Clauses pursuant to the Commission Decision C (2010) 593, a copy of which is available to you upon request to the study doctor.

Transfers Out Of Your Country

For this clinical study, your child's data will be transferred to countries outside of «country» and «this requires your consent / the recipients of your child's personal data shall abide by privacy principles at least equivalent to the legal requirements for personal data processing in your country».

How Long We Will Keep The Information

Your child's personal data will be retained for «[x] years/a period determined by applicable laws and regulatory requirements, which could be for the entire life of the study drug or a longer period».

Your Child's Rights

The consent you provide today does not have an expiration date and will last unless and until you cancel it. You may withdraw your consent for us to use and share your child's personal data at any time by notifying the study doctor in writing. You can do this by sending written notice to the study doctor at the address in **Section 19 (page 13)** *EEA only:* or the sponsor's Data Protection Officer at sar@thedpo.co.uk. If you withdraw your consent for processing, your child will not be able to stay in this study. When you withdraw your consent, no new personal data about your child will be collected after that date but a minimum level of information may need to be retained for safety purposes or other important legal reasons in the public interest. This is so that you can be contacted if an adverse effect is discovered after your child has withdrawn from the study. Please be aware that withdrawing your child from the study will not be considered to be a withdrawal of consent to process your child's personal data unless you also tell us to stop processing your child's personal data. *US only:* However, the personal data that has already been collected may still be used and given to others.

You and your child have the right to see and make copies of their own medical records or any personal data held by the study doctor or sponsor at any time. *US only:* You are agreeing, however, by signing this form, not to ask to see or copy your child's study data until the sponsor has completed the work related to this study. When the study is over, you may ask on behalf of your child to see your child's study data.

You and your child also have the right to rectify, request the deletion or object to the processing of any of your child's personal data that is held by the study doctor or sponsor at any time. You can do so by contacting the study doctor in writing at the address in **Section 19 (page 13)** or the sponsor's Data Protection Officer at sar@thedpo.co.uk.

In addition, if you are unhappy with how your child's personal data is being processed, you have the right to lodge a complaint with your country's data protection supervisory authority, «local regulatory authority and its contact details».

Please be aware that if you decide not to provide your consent for processing your child's personal data, your child will not be able to be in the study. However, your consent for future research to be performed with your child's personal data is optional.

Consent to Data Processing

Please tick the options below as appropriate.

☐ I have read this form and its contents were explained to me. My questions have been answered. I voluntarily agree to allow the study staff to collect, use, and share my child's personal data in the manner and for the study, as outlined above. I will receive a signed copy of this form for my records.

☐ I also agree for my child's information to be used in future research in accordance with the safeguards outlined above and understand that because the future research needs have not yet been defined, the methodology of that research cannot be explained to me at this time.

APAC/ROW countries that require consent for transfers only:

☐ I agree to the transfer of my child's personal data outside of our country of origin, subject to appropriate safeguards and protections.

Signature of the study participant's parent/guardian	Date<br>Parent/guardian initials: ____________
Signature of the study participant's parent/guardian	Date<br>Parent/guardian initials: ____________
Signature of the investigator	Date
